# Spatial patterns of the bird-related sentiment index and its nonlinear environmental associations: Evidence from social media and explainable machine learning

**DOI:** 10.1371/journal.pone.0360060

**Published:** 2026-11-10

**Authors:** Jue Wang, Peiyi Du, Yao Zhao, Sihui Chen, Jiaxin Liao, Shunhe Chen

**Affiliations:** College of Landscape Architecture and Arts, Fujian Agriculture and Forestry University, Fuzhou, Fujian, China; Zhejiang Agriculture and Forestry University: Zhejiang A and F University, CHINA

## Abstract

As cities become denser and more artificial, encounters with birds are closely tied to how residents perceive urban nature. However, evidence on the spatial patterns and environmental correlates of bird-related emotions remains limited. Taking Shanghai as a case study, this study cleaned 4,142 bird-related Weibo check-in comments from 2022 to 2024. A deep-learning sentiment model was used to construct a bird-related sentiment index, which was linked to 500 m × 500 m grids and examined with climatic, ecological, landscape, and human-activity indicators. Spatial analysis and interpretable machine learning were applied to identify emotional clusters, key environmental predictors, and spatial differences in model contributions. Results show that: (1) bird-related sentiment is generally positive but spatially uneven, with more continuous positive areas in suburbs and waterfronts and more fragmented responses in the urban core; (2) higher heat exposure and air pollution are associated with lower predicted sentiment scores, whereas vegetation, green coverage, and proximity to green and blue spaces correspond to higher predicted scores; (3) central districts show stronger negative model contributions from heat and pollution, while suburbs and waterfront corridors are associated with more positive predicted sentiment patterns and may provide settings where bird habitats and positive human experiences overlap.

## Introduction

In the high-density living environments of megacities, people’s interactions with nature increasingly occur within their everyday activity spaces rather than through occasional long-distance travel [1]. Access to and use of urban nature are not shaped by a single determinant. Rather, they arise from multiple socio-ecological drivers, which contribute to marked within-city differences in the health effects of nature exposure [2]. A growing body of evidence shows that exposure to different forms of urban nature—such as parks, urban forests, and waterfronts—can yield short-term benefits, including reduced stress and anxiety and improved overall mental health [3]. However, this association is often nonlinear. As exposure increases, marginal psychological benefits tend to diminish and eventually plateau [4,5]. The health effects of urban nature are not explained by vegetation cover alone. Increasingly, public health frameworks explicitly incorporate the “species” dimension of biodiversity [6,7]. Using postal-code–based spatial units across multiple Canadian metropolitan regions, studies have found that greater bird and tree species diversity is significantly associated with better self-rated mental health. This association remains after adjustment for demographic and socioeconomic factors [8]. These findings suggest that urban biodiversity is relevant to ecological conservation and may also shape residents’ emotions and well-being through mechanisms grounded in everyday perception and experience [9,10].

In urban biodiversity contexts, birds are highly perceptible to the public because they can be both seen and heard in daily life [11]. Chen et al.(2025) and Yi et al.(2025) treat birdsong and avian activity as integral elements of urban soundscapes. They show that interactions between birdsong and traffic noise can substantially shift perceived soundscape pleasantness and landscape quality [12,13]. Gilmour et al.(2024) further report that natural soundscapes can facilitate emotional restoration even under noise pollution, and that these benefits can be quantified [14]. Multisensory in situ randomized experiments also suggest that soundscape and landscape quality jointly shape stress recovery, with soundscape effects potentially stronger [15]. Therefore, studies of bird-related emotions may be poorly explained by macro-scale vegetation metrics or generic emotion frameworks alone, particularly when accounting for spatial heterogeneity within cities. Bardhan et al.(2024) show that street-level visibility of greenery and water is associated with mental health. Moreover, across neighborhoods and environ-mental contexts, differences in visual exposure can produce distinct emotional responses. Collectively, these studies indicate that within-city differences are associated with the amount of vegetation, the quality of exposure, and the ways people interact with nature. Such differences require more fine-grained spatial and emotional indicators for adequate explanation [16]. Xu et al.(2024) raise the question of why, within the same urban environment, certain areas are more likely to trigger positive bird-related perceptual experiences, while specific environmental stressors may undermine such experiences [17]. Translating this notion of “bird perceptual experience” into measurable emotional evidence requires simultaneously meeting two conditions: extensive spatial coverage and fine-scale interpretability [18]. Compared with questionnaires and on-site experiments, geotagged social media texts can capture intra-urban emotional spatial differences at a finer resolution, while also facilitating coverage across multiple time periods and locations of everyday expression [19]. National-scale studies based on Weibo have already demonstrated that extreme temperatures causally reduce public emotional well-being and have revealed inter-city differences in exposure and vulnerability [20]. Subsequent research has further shown that the relationship between emotions and urban temperature is often nonlinear, and that the effects of landscape elements vary across city hierarchies and gender groups [21]. However, most existing studies remain focused on generalized emotional indicators and macro-scale two-dimensional exposure representations, with relatively limited discussion of spatial differentiation under bird-specific thematic contexts. In particular, three-dimensional perceptual variables at the street level and social media data have not yet been fully integrated within a unified analytical framework, making it difficult to explain why certain areas within the same city are more sensitive or more responsive to avian cues [19]. Another challenge lies in how nature exposure is measured. Remote-sensing vegetation indices (e.g., NDVI) capture city-scale vegetation cover but may not represent the greenery residents perceive at street level. To address this mismatch, recent studies use street-view imagery to quantify pedestrian-visible greenery, for example via the Green View Index (GVI). This approach better approximates everyday visual exposure and supports more actionable planning interpretation [22,23]. Review evidence suggests that deep learning is shifting research on urban environments, emotional perception, and mental health from coarse coverage metrics toward finer-scale visual cues and spatial elements. However, key challenges remain, including improving interpretability and translating model outputs into planning practice [24].

Mechanistically, associations between urban environmental factors and emotional responses often involve thresholds, nonlinearities, and interactions. Therefore, simple linear correlations are insufficient for identifying actionable ranges or meaningful combinations of drivers [25]. Wang et al.(2025) show that explainable models combining XGBoost and SHAP can quantify each variable’s contribution while also indicating effect direction. This enables identification of nonlinear environmental influences on emotional perception [26]. In spatial analyses, treating geographic coordinates as ordinary predictors often leads to the generic conclusion that “location matters,” without revealing why. Such models also struggle to separate effects attributable to location itself from those arising through location–environment interactions [27]. Consequently, explaining spatial heterogeneity requires an explicit attribution framework that identifies the key factors and reveals where their effects are strongest and how they vary across urban areas [28,29]. Li et al.(2024) extend GeoShapley by explicitly incorporating geographic location into explainable analysis. Using Shapley values, they decompose the independent contribution of spatial position and its interactions with other variables. This framework yields spatial effect attributions that are more comparable across locations and more interpretable within machine learning models [30].

As interest grows in the relationship between urban nature experiences and residents’ emotions and well-being, urban ecological research has largely focused on bird diversity, habitat conditions, and their spatial patterns. Public emotional responses associated with birds in everyday urban settings, however, remain less systematically quantified, and the environmental conditions associated with their within-city variation are still insufficiently understood. In particular, thermal conditions, air pollution, access to blue–green spaces, and street-level perceptual environments may act together and may exhibit nonlinear and spatially heterogeneous associations.

Against this background, this study addresses three research questions. RQ1: How is the bird-related sentiment index distributed across Shanghai, and does it exhibit significant spatial clustering? We hypothesize that bird-related sentiment is spatially structured rather than randomly distributed, with significant positive spatial autocorrelation across neighboring areas. RQ2: Which climatic, ecological, landscape, and human-activity factors are most strongly associated with variation in the bird-related sentiment index, and do these associations exhibit nonlinear or threshold patterns? Based on previous evidence, we expect higher thermal stress and air pollution to be associated with lower predicted sentiment, whereas greater vegetation exposure and better proximity to green and blue spaces are expected to be associated with higher predicted sentiment. RQ3: Do the model contributions of these environmental factors vary across urban space? Given the marked spatial differences in urban form and environmental exposure across Shanghai, we expect the magnitude and direction of major environmental contributions to vary among locations.

To address these questions, we integrate geotagged Weibo texts, street-view-derived perceptual indicators, and multi-source environmental data within a 500 m × 500 m grid framework. Spatial analysis is first used to characterize the overall distribution of bird-related sentiment. XGBoost and SHAP are then applied to identify major environmental associations and their nonlinear patterns, followed by GeoShapley-based spatial contribution analysis to examine geographic variation in model contributions. This framework links spatial patterns, environmental associations, and spatial heterogeneity, providing evidence for biodiversity-friendly urban regeneration and blue–green space management.

## Methods

### Study area

The study area is Shanghai (30°40′–31°53′ N, 120°52′–122°12′ E), situated on the coastal plain at the mouth of the Yangtze River (Fig 1). The city contains dense river networks and coastal wetlands interwoven with high-intensity built-up areas. As a key node along the East Asian–Australasian Flyway, Shanghai’s estuarine wetlands (e.g., Chongming Dongtan) provide critical habitat for migratory birds, functioning as stopover, foraging, and breeding sites. Habitat quality is closely associated with land-use change and landscape fragmentation [31]. At the urban scale, Shanghai retains a relatively rich baseline of avian diversity. At the same time, the built environment and its spatial configuration strongly shape bird diversity patterns [32]. Behavioral evidence suggests that bird-related outdoor activities, such as birdwatching, can enhance subjective well-being and reduce psychological distress [33]. This supports extending bird-focused research to public emotional responses. Accordingly, we use Shanghai as the case study to examine nonlinear relationships between bird-related emotional responses and landscape factors. To ensure spatial consistency, all datasets were projected to WGS 84 / UTM Zone 51N. We then created a regular 500 m × 500 m grid in ArcGIS as the basic analytical unit. A 500 m × 500 m grid was selected to balance spatial detail and sample adequacy. This resolution is fine enough to capture intra-urban differences in street-level environmental exposure, blue–green accessibility, and built-environment intensity, while avoiding excessive fragmentation of Weibo observations at a very small spatial scale. During spatial matching, 4,011 of the 4,142 valid Weibo comments were successfully assigned to the 500 m × 500 m grid system, with a matching rate of 96.84%. The remaining 131 comments were not included in the grid-based statistics, mainly because of boundary differences after the exclusion of water areas or minor coordinate-position discrepancies. Based on the spatial join results, 1,280 grid cells contained at least one valid Weibo comment. Among these occupied grids, the average number of comments was 3.13; 310 grids contained no fewer than three comments, and 157 grids contained no fewer than five comments. The grid-level sample distribution is reported in S1 Table in S1 File. For grids with valid comments, the bird-related sentiment index was calculated as the mean sentiment score of comments within each grid. Grids without comments were not assigned artificial zero or neutral sentiment values. Emotional indices and environmental indicators were aggregated and linked at the grid level, yielding a unified spatial framework for subsequent analyses

**Fig 1:**
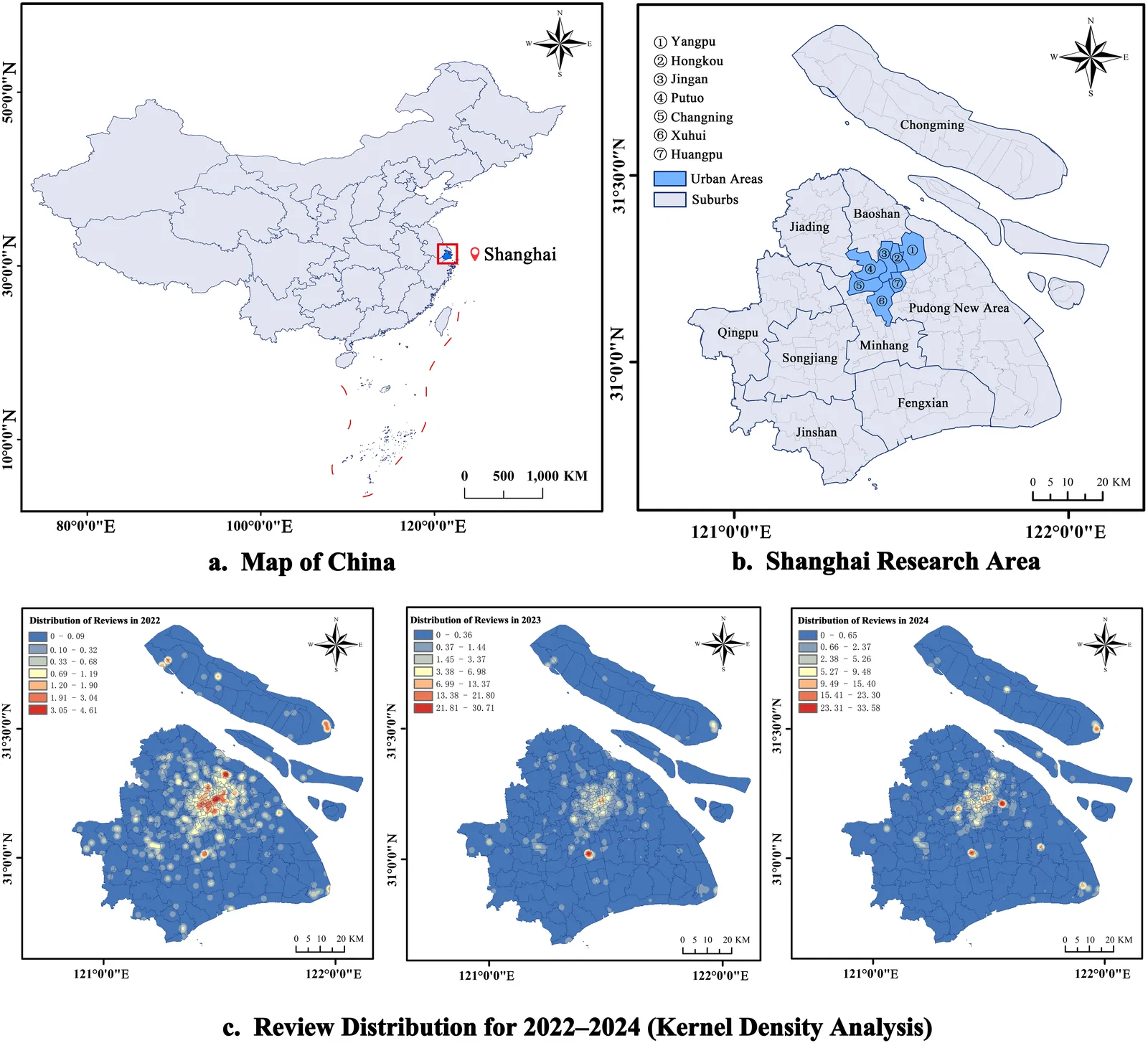
Study area. Note: The national location map in panel (a) was based on the standard map with approval number GS(2022)4308. The Shanghai administrative boundary in panels (b) and (c) was checked against the Shanghai administrative division standard map with approval number Hu S(2025)049. The base map boundaries were not modified, and the thematic layers were generated by the authors using processed analytical data. URLs: http://bzdt.ch.mnr.gov.cn/; https://shanghai.tianditu.gov.cn/map/views/standardMap.html.

### Analytical workflow

The analytical workflow comprised three main stages (Fig 2). First, bird-related Weibo check-in comments posted in Shanghai from 2022 to 2024 were collected and screened. Comment-level sentiment scores were then derived using the ERNIE-assisted LSTM–CNN model and aggregated within 500 m × 500 m grid cells to construct the bird-related sentiment index. Previous studies have shown that geotagged social media texts can be used to characterize spatiotemporal variations in urban emotions and support their integration with built-environment variables [19,34]. Second, multi-source environmental data covering climatic, ecological, landscape, and human-activity conditions were assembled. Street-level perceptual indicators were extracted from street-view imagery using Mask2Former, and all environmental variables were aggregated to the same grid scale. Third, Kernel Density Estimation and Global Moran’s I were used to characterize spatial distributions and spatial autocorrelation, while Spearman’s rank correlation provided a descriptive assessment of bivariate associations. XGBoost and SHAP (Shapley Additive Explanations) were then used to identify major environmental factors and their nonlinear associations [28,35], followed by GeoShapley, a spatially explicit Shapley-based interpretation approach, to examine geographic variation in predictor-specific model contributions [30]. Detailed data-processing and analytical procedures are described in the following sections.

**Fig 2:**
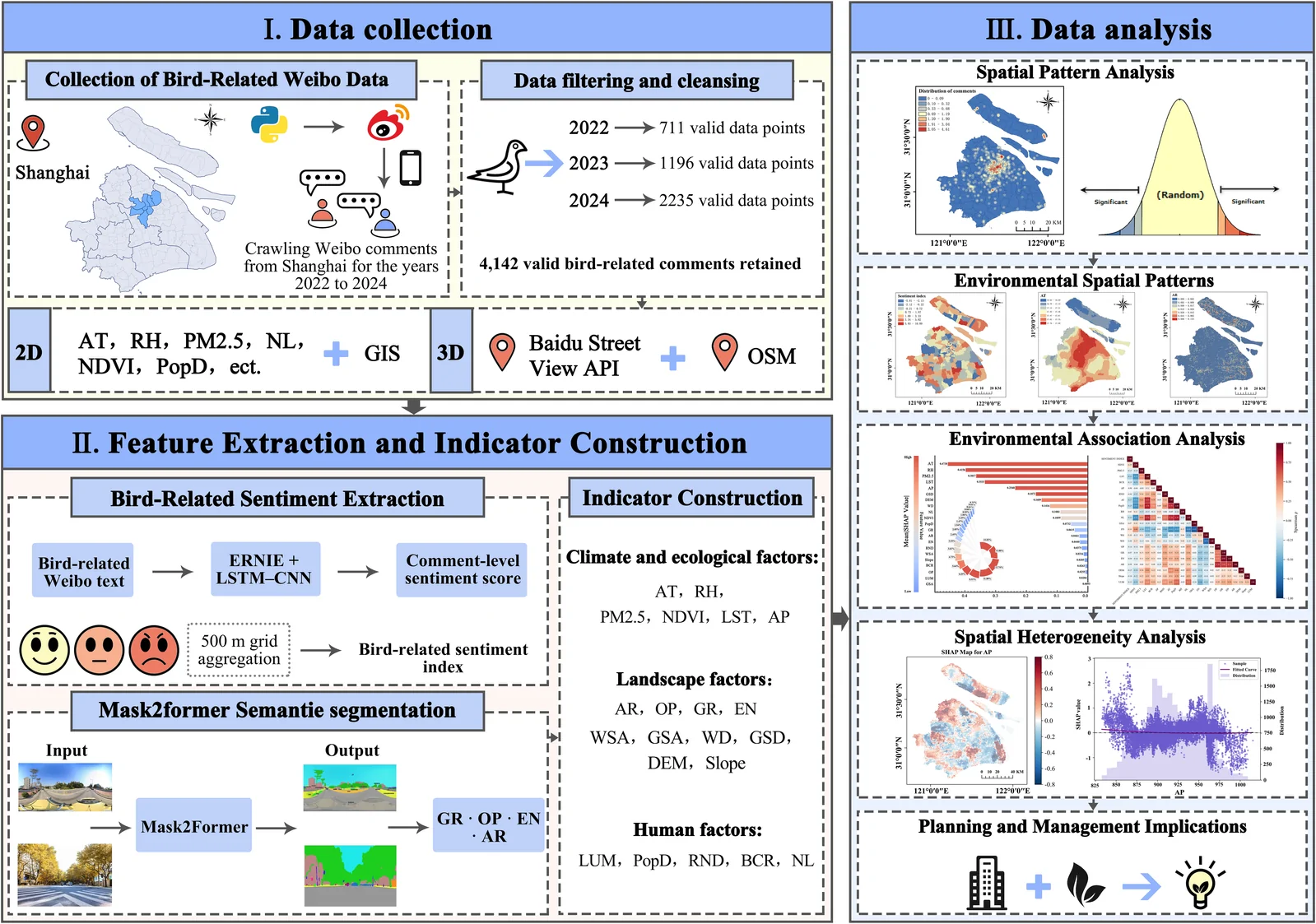
Analytical workflow of the study. Note: The Shanghai administrative boundary shown in this figure was checked against the Shanghai administrative division standard map with approval number Hu S(2025)049 (https://shanghai.tianditu.gov.cn/map/views/standardMap.html). The workflow, thematic layers, model outputs, and analytical diagrams were generated by the authors using processed analytical data.

### Data sources and processing

#### Collection of bird-related weibo check-in data.

Weibo is a widely used social media platform in China that allows users to post textual content and attach location information through its check-in function. In this study, “Weibo check-in comments” refer to user-generated textual content posted together with location-based check-in information. We collected bird-related Weibo check-in comments posted in Shanghai between January 1, 2022, and December 31, 2024, and used them as the textual source for constructing the bird-related sentiment index. To reduce the potential positive-selection bias caused by using only appreciation-oriented keywords such as “birdwatching” or “birdsong,” we adopted a broader keyword strategy. The retrieval terms included general and neutral bird-related expressions, such as “bird,” “wild bird,” “bird flock,” and “birds in the park,” as well as experience-oriented expressions such as “birdwatching” and “birdsong.” In addition, we considered potentially negative bird-related expressions, including terms associated with bird noise, droppings, nuisance, and disturbance, in order to capture a wider range of public perceptions rather than only positive recreational experiences. We retained only records with valid check-in coordinates so that each post could be assigned to a specific street location. The dataset was then deduplicated and cleaned. Advertisements, irrelevant content, emojis, posts without clear evidence of direct nature exposure, purely informational reposts, pet-bird discussions, and internet slang were removed to reduce semantic noise from non-experiential contexts. Ultimately, 4,142 valid comments were obtained, including 711 from 2022, 1,196 from 2023, and 2,235 from 2024. The detailed data-flow process, screening rules, keyword categories, and yearly sample changes are provided in S2 Table in S1 File. Briefly, the initial keyword retrieval yielded 48,595 Weibo check-in comments from 2022 to 2024, including 10,044 posts in 2022, 13,220 in 2023, and 25,331 in 2024. After field checking, duplicate checking, coordinate validation, and manual thematic relevance screening, 4,142 valid comments were retained for analysis. It should be noted that Weibo check-in comments represent expressions from active Weibo users rather than the entire urban population. Therefore, the bird-related sentiment index constructed in this study reflects the spatial sentiment patterns of users who posted bird-related check-in comments, and should not be interpreted as a direct measure of the perceptions of all residents in Shanghai.

### Bird-related sentiment index construction using an LSTM–CNN model

Sentiment analysis was used to distinguish positive, neutral, and negative emotional expressions in bird-related Weibo check-in texts and to convert these expressions into a quantitative measure that could be compared across space. Check-in frequency alone indicates where and how often bird-related discussions occur, but it does not reveal whether users expressed positive or negative responses to those experiences. Sentiment extraction was therefore necessary to construct the bird-related sentiment index used in the subsequent spatial and environmental analyses. To identify sentiment polarity in bird-related Weibo comments, we first use the pretrained language model ERNIE(https://github.com/PaddlePaddle/LARK/tree/develop/ERNIE) to generate contextual text embeddings. These embeddings improve the representation of contextual information in short Chinese texts. On this basis, a Long Short-Term Memory (LSTM) network is used to encode the text sequences, learning long-range dependencies and semantic relations such as negation, contrast, and progression, so that emotional orientation remains coherent across ex-tended contexts. Subsequently, a Convolutional Neural Network (CNN) is applied to the sequence of LSTM hidden states. Through one-dimensional convolution and pooling operations, the CNN extracts the most discriminative key fragments and compositional features within local windows, enabling a complementary integration of global contextual understanding and local evidence enhancement. Compared with traditional classifiers such as support vector machines (SVM) and Naive Bayes—which rely heavily on manual feature engineering and vocabulary coverage—this end-to-end architecture better reflects the shift in Chinese sentiment analysis from handcrafted features to deep representation learning [36]. The contextual semantic representations produced by ERNIE improve the encoding of short-text contexts and, in coordination with the sequence modeling and convolutional modules, enhance sentiment classification performance [37]. Within bird-related corpora, emotional expressions often exhibit class imbalance and stylistic diversity. The combination of LSTM-based sequence modeling and CNN-based local evidence reinforcement helps improve recall for minority emotion classes and overall classification stability. To further validate the model on the bird-related Weibo corpus, we constructed a manually annotated validation subset of 500 comments from the final cleaned dataset. The comments were labeled into three sentiment classes: negative, neutral, and positive. For classification-based validation, model-generated comment-level sentiment scores were converted into three classes: scores below −1 were classified as negative, scores from −1 to 1 as neutral, and scores above 1 as positive. Model performance was evaluated using precision, recall, F1-score, macro average, weighted average, overall accuracy, and a confusion matrix. The validation results are reported in S3 Table in S1 File, and the confusion matrix is provided in S4 Table in S1 File. The model achieved an overall accuracy of 0.898 and a weighted average F1-score of 0.898, indicating reliable performance on the bird-related Weibo sentiment classification task. The validated comment-level sentiment scores were then used to construct the bird-related sentiment index for subsequent spatial and machine-learning analyses. After model validation, the comment-level sentiment scores were aggregated to the grid scale. Each valid Weibo comment was assigned to a 500 m × 500 m grid cell according to its check-in coordinates. The grid-level bird-related sentiment index was calculated as the arithmetic mean of all comment-level sentiment scores within each grid. Grids with at least one valid comment were included, whereas grids without valid comments were excluded from sentiment-based spatial analysis and machine-learning modeling. We did not set a minimum comment threshold for grid inclusion, because doing so would remove observations from low-activity areas and reduce the representation of spatially dispersed bird-related experiences. The 2022–2024 data were pooled for the main analysis, and each comment was given equal weight. At the grid level, each occupied 500 m × 500 m cell was treated as a single observation in subsequent modeling, regardless of the number of comments contained within that cell. Consequently, grids with more comments did not receive greater model weight or enter the model multiple times. We recognize, however, that mean sentiment values derived from sparsely sampled grids may be less stable than those based on a larger number of comments. To retain spatially dispersed bird-related experiences, no minimum comment threshold was imposed. The distribution of comment counts across occupied grids is reported in S1 Table in S1 File to make differences in sampling density transparent. Since the sentiment scores were already bounded between −10 and 10 and irrelevant posts had been removed during manual screening, no additional outlier adjustment was applied. The resulting bird-related sentiment index was used as the dependent variable in the subsequent spatial and machine-learning analyses. The sentiment analysis was conducted at the level of the overall bird-related corpus, and specific bird species or lexical expressions were not modeled as separate explanatory categories. Accordingly, the results represent general patterns of bird-related sentiment and its environmental associations, and should not be interpreted as species-specific or phrase-specific emotional effects. This modeling strategy is also consistent with recent applications of machine learning in urban bird-related environmental research [38].

### Quantification and extraction of environmental factor data

To capture the ecological basis and enhance the interpretability of relationships between bird-related emotions and landscape environments, this study—drawing on recent research on urban bird habitat preferences and built-environment thresholds—constructed an environmental factor system comprising 21 variables across three dimensions: climatic–ecological, environmental–landscape, and human activity (S5 Table in S1 File). Previous studies have shown that factors such as vegetation cover and height, the presence of water bodies, and humidity are closely associated with urban bird diversity and often exhibit nonlinear threshold patterns, while building height and density are often associated with lower avian activity [32,39]. Among the variables, AR, OP, GR, and EN represent three-dimensional perceptual indicators. Street-view data collection and processing were completed in March 2025. The acquisition years of Baidu Street View imagery were predominantly 2022–2023, closely aligned with the study period and therefore suitable for representing street-level spatial characteristics during the analysis timeframe. Following an existing Shanghai street-view semantic segmentation workflow [40], sampling points were generated at 50 m intervals along the OpenStreetMap (OSM) road network in ArcMap, and street-view images were obtained through the Baidu Street View API. At each sampling point, four fixed viewing directions were collected, namely 0°, 90°, 180°, and 270°. In total, the dataset contained 471,839 street-view sampling points and 1,887,356 street-view images. Before semantic segmentation, inaccessible, duplicated, severely blurred, heavily occluded, and obviously abnormal-view images were excluded to ensure the reliability of subsequent streetscape indicator extraction. Multi-directional images were stitched into panoramic views using OpenCV and then processed using a Mask2Former semantic segmentation model trained on the Mapillary Vistas dataset. The segmentation outputs were used to extract the proportional coverage of greenery, sky, buildings, road surfaces, and water-related elements, which were then used to calculate Greenness, Openness, Enclosure, and Aquatic Rate. A summary of the street-view image collection, sampling design, image quantity, acquisition years, quality-control screening, and semantic segmentation processing is provided in S6 Table in S1 File. All remaining variables were aggregated at the grid scale using remote sensing and spatial datasets. Given empirical evidence that bird-related nature exposure is associated with emotional well-being [41], these environmental factors were spatially aligned with the Weibo-based bird-related sentiment within 500 m × 500 m grid cells, projected to WGS 84 / UTM Zone 51N, and summarized as grid-level mean values for subsequent model training and explainable analysis.

### Street-view indicator extraction using mask2Former

To obtain street-level environmental cues at the same spatial scale as “bird-related emotions,” this study constructs an environmental visual information extraction workflow based on street-view imagery [42]. Street-view data directly capture pedestrian-visible characteristics such as greenery exposure and spatial openness, making them well-suited for spatial alignment with social media–derived emotional measures [22]. Specifically, sampling points were deployed along the road network to acquire multi-directional street-view images, which were stitched into panoramic views to reduce information loss associated with single-view perspectives [43]. Mask2Former was then applied for semantic segmentation, generating pixel-level masks for categories including vegetation. The proportional area of each category was subsequently converted into quantitative indicators [44]. In recent years, mask-based Transformer segmentation methods have demonstrated strong boundary stability and generalization performance in high-resolution urban scenes, providing a robust foundation for large-scale extraction of environmental elements [45,46]. Based on this workflow, we derived four three-dimensional perceptual indicators—Greenness (GR), Openness (OP), Enclosure (EN), and Aquatic Rate (AR). We aggregated these indicators, together with other two-dimensional environmental variables, within 500 m × 500 m grid cells to create a unified input for subsequent nonlinear modeling. Prior work suggests that standardized processing and reuse of large-scale street-view datasets can improve workflow traceability and reproducibility [47].

### Data analysis

#### Kernel density analysis based on ArcMap.

Kernel Density Estimation (KDE) is used to measure the local intensity of spatial events by transforming discrete point data into a continuous density surface, thereby revealing spatial patterns of hotspots and cold spots [48]. In this study, KDE is primarily applied to characterize the degree of spatial clustering of bird-related Weibo check-ins across the urban area. Each valid bird-related Weibo check-in comment was treated as a spatial point, and its neighborhood contribution was calculated within a specified search radius to derive the spatial density of bird-related check-in comments. The resulting density raster was used to identify areas where bird-related Weibo discussions were relatively concentrated and to provide spatial context for subsequent comparisons with landscape and environmental factors. In practice, the Kernel Density tool in ArcMap was used to generate density layers, and density surfaces corresponding to different years or phases were compared at the same spatial scale to examine whether bird-related emotional perception exhibits stable spatial clusters and how these clusters evolve over time. The calculation formula is as follows:


$$\begin{array}{c} \text{Density =}\frac{\text{1}}{(radius{)}^{\text{2}}}\sum_{i=1}^{n}\left[\frac{\text{3}}{\pi }\cdot pop_{i}{\left(1-(\frac{disti}{radius}{)}^{\text{2}}\right)}^{\text{2}}\right] \end{array}$$ (1)

Here, i=1,...,n denotes point data assigned from built-environment indicator datasets; $pop_{i}$ represents an optional parameter corresponding to the attribute value assigned to point i; and $dist_{i}$denotes the distance between point i and location (x,y).

### Global Moran’s I spatial autocorrelation

Global Moran’s I was used to assess whether an individual spatial variable exhibited overall spatial autocorrelation across the study area, that is, whether neighboring spatial units tended to contain similar or dissimilar values [49]. In this study, Global Moran’s I was calculated separately for the bird-related sentiment index and for each environmental variable. The resulting statistics therefore describe the spatial clustering of each variable itself, without controlling for other environmental factors, and were not used to identify independent or combined environmental causes of clustering in bird-related sentiment. Associations between environmental factors and bird-related sentiment, as well as their spatial variation, were examined separately using Spearman’s rank correlation, XGBoost–SHAP, and GeoShapley-based spatial contribution analysis. The calculation formula is as follows:


$$\begin{array}{c} I=\frac{n\sum_{i=\text{1}}^{n}\sum_{j=\text{1}}^{n}w_{ij}(x_{i}-\overset{―}{x})(x_{j}-\overset{―}{x})}{\sum_{i=\text{1}}^{n}\sum_{j=\text{1}}^{n}w_{ij}\sum_{i=\text{1}}^{n}{\left(x_{i}-\overset{―}{x}\right)}^{\text{2}}} \end{array}$$ (2)

In spatial statistical analysis, n represents the total number of spatial units, while $x_{i}$ and $x_{j}$ correspond to the observed attributes of the i and j units, respectively. Together with the global mean $\overset{―}{x}$ and the spatial weight matrix element $w_{ij}\;$, which quantifies the adjacency or interaction strength between spatial units i and j, Moran’s I measures the degree of spatial association. The value of Moran’s I is typically bounded within the interval [−1,1]. When I>0, positive spatial autocorrelation is indicated: units with similar values tend to cluster geographically. When I<0, negative spatial autocorrelation is indicated, characterized by a dispersed (checkerboard-like) pattern in which high and low values are interspersed. When I=0, the spatial pattern approximates randomness, indicating little to no spatial dependence.

### Spearman’s rank correlation coefficient

After constructing the grid-level bird-related sentiment index, Spearman’s rank correlation was used to examine bivariate associations between the index and the 21 environmental variables at the same 500 m × 500 m spatial scale. This nonparametric method does not require normally distributed variables and is suitable for describing monotonic relationships, providing a descriptive baseline for the subsequent nonlinear machine-learning analysis [50]. Pairwise correlations among environmental predictors were also calculated to identify potentially strong intercorrelations. The Spearman rank correlation is defined as follows:


$$\begin{array}{c} \rho \text{=1-}\frac{\text{6}\sum {\text{d}}_{\text{i}}^{\text{2}}}{\text{n}\left(n^{\text{2}}-\text{1}\right)} \end{array}$$ (3)

Here, ${\text{d}}_{\text{i}}$ denotes the rank difference of the i pair of observations, and n represents the sample size. The pairwise Spearman correlation coefficients ρ calculated among all variables were assembled into a correlation matrix and visualized as a heatmap to illustrate the direction (positive or negative) and strength of the associations between the bird-related sentiment index and environmental factors.

### XGBoost modeling and SHAP-based interpretability analysis

To evaluate whether the environmental predictors can explain the bird-related sentiment index, we compared three gradient-boosting models—XGBoost, CatBoost, and LightGBM—using an 80% training set and a 20% testing set. A fixed random seed (random_state = 123) was used to ensure reproducibility, and the same training–testing split was applied to all three models. Hyperparameter tuning was conducted on the training set using 5-fold cross-validation, with RMSE used as the main optimization criterion. The final hyperparameters of the three gradient-boosting models are reported in S7 Table in S1 File. Before model fitting, correlations among predictors were examined using the Spearman correlation matrix. Several variable pairs showed relatively high correlations, including GR–EN, LST–PopD, and PopD–NL. Because these variables represent conceptually distinct aspects of street morphology, thermal conditions, and human activity, and because the analysis relied on tree-based gradient-boosting models, variables were not removed solely on the basis of pairwise correlation. We note, however, that highly correlated predictors may share model attribution during interpretation. Accordingly, SHAP values are interpreted as predictor contributions within the fitted model rather than as independent or causal effects of individual variables. The correlation structure among predictors is shown in Fig 5. Model performance was evaluated using R² and RMSE on the same scale of the bird-related sentiment index. The model performance comparison is shown in Table 1. XGBoost achieved the highest testing R² of 0.7557 and the lowest testing RMSE of 1.3452. Its 5-fold CV RMSE was 1.4209, which was lower than those of CatBoost and LightGBM. LightGBM also showed relatively strong performance, with a testing R² of 0.7383 and a testing RMSE of 1.3923, whereas CatBoost showed lower overall performance. Considering testing-set explanatory performance, prediction error, cross-validation results, and the maturity of its interpretation framework, XGBoost was selected as the core modeling approach for subsequent SHAP and GeoShapley analyses. XGBoost can robustly capture nonlinear responses in settings with high-dimensional predictors, heterogeneous scales, and complex interactions. It has been widely used to identify driving factors and assess risks in urban environmental research [51]. To improve interpretability, we use SHAP (Shapley Additive Explanations) to decompose model outputs. Based on Shapley value theory, SHAP quantifies each variable’s marginal contribution to the prediction and provides both contribution direction and an importance ranking. SHAP also helps identify threshold effects and interactions, providing clearer insight into how landscape and environmental variables relate to variation in bird-related emotional responses. The SHAP formulation is given as follows:

**Table 1:** Model performance comparison.

Metric	XGBoost	CatBoost	LightGBM
**Training set RMSE**	0.8924	1.5360	1.0474
**Testing set RMSE**	1.3452	1.6385	1.3923
**Training set R²**	0.8923	0.6810	0.8517
**Testing set R^2^**	0.7557	0.6375	0.7383
**5-fold CV RMSE**	1.4209	1.6677	1.4421


$$\begin{array}{c} g\left(z^{\prime }\right)=\phi_{\text{0}}+\sum_{j=1}^{M}\phi_{j}{z^{\prime }}_{j} \end{array}$$ (4)

Here, g(z′) denotes the model’s predicted value of the bird-related sentiment index, $\phi_{\text{0}}$ represents the baseline output, $\phi_{j}$is the SHAP value of the j variable,$z_{j}^{\prime \;}$ indicates whether a given feature is present, and M denotes the total number of features.

### GeoShapley-based spatial contribution analysis

To further examine the spatial heterogeneity of model explanations, we applied a GeoShapley-based spatial contribution analysis after fitting the XGBoost model. Following previous studies that use SHAP-explained XGBoost models to extract spatial effects from geo-referenced data [30], this study treated each 500 m × 500 m grid cell as a spatial observation unit and used its grid geometry as the spatial carrier for visualizing local model contributions. This approach is also consistent with recent applications in which GeoSHAPLEY outputs are presented as spatialized SHAP values to reveal geographic heterogeneity in environmental contributions. The analysis was implemented in Python using GeoPandas, XGBoost, scikit-learn, and SHAP. The GeoJSON grid file contained the grid geometry, the bird-related sentiment index, and environmental predictors. After the XGBoost model was fitted, SHAP values were calculated for all grid samples. For each environmental predictor, the corresponding local SHAP values were joined back to the 500 m × 500 m grid geometries and visualized as spatial contribution maps. In this study, spatial information was therefore used to locate and visualize the geographic variation of predictor-specific SHAP contributions, rather than to estimate an independent coordinate-based causal effect.

The GeoShapley-based outputs were interpreted as spatially explicit model contributions to the predicted bird-related sentiment index. Positive values indicate that a predictor increases the model prediction relative to the baseline prediction, whereas negative values indicate that a predictor lowers the model prediction. To support interpretation, the spatial contribution maps were compared with the global SHAP importance results, PDP patterns, and the observed spatial distribution of the bird-related sentiment index. The additive decomposition formulation is given as follows:


$$\begin{array}{c} \phi_{i}\text{=}\sum_{S\subseteq F\backslash \left\{i\right\}}\frac{\left|S\right|!(|F|-|S|-1)}{\left|F\right|!}\left[f\left(S\cup \left\{i\right\})-f(S\right)\right] \end{array}$$ (5)

Here, $\phi_{i}$ denotes the Shapley-based contribution value of feature i;S represents a feature subset that excludes feature i;Fis the full set of features; |S|denotes the size of subsetS; |F| denotes the size of feature setF;f(S) is the model prediction using only subsetS; and f(S∪{i})represents the prediction after adding feature i.

## Results

### Spatial distribution of the bird-related sentiment index

Based on LSTM–CNN–based sentiment identification of 4,142 Weibo check-in comments, the mean value of the bird-related sentiment index is 1.64, with a scaled range from −10–10, indicating an overall mildly positive tendency accompanied by substantial variability (Fig 3a). The samples are widely dispersed across both positive and negative ends of the spectrum, with occurrences of both high and low values, suggesting pronounced differentiation in public emotional experiences of the landscape environment rather than concentration around a single intermediate range. Spatially, the bird-related sentiment index shows pronounced patchiness and heterogeneity (Fig 3b). Chongming District is dominated by medium-to-high values (≈1.88–10.00), forming locally continuous clusters of positive emotion. In contrast, central districts (Huangpu, Jing’an, Xuhui, Changning, Putuo, Hongkou, and Yangpu) show interwoven high and low values. Medium–low (≈−2.12 to 1.87) and medium–high patches alternate over short distances, producing a finely fragmented mosaic. Spatial differentiation becomes clearer toward the suburban periphery. In western Shanghai, Qingpu and Songjiang exhibit relatively concentrated low-value clusters (≈−5.81 to −2.13). Parts of northern Jiading and adjacent Baoshan also show clusters of medium–low values. In eastern Pudong, a banded contrast emerges. The central zone tends toward lower values, whereas the southeastern coast and some waterfront edges more often show medium–high to high clusters. Overall, Shanghai exhibits multiple hotspots and cold spots in the bird-related sentiment index. Fragmentation is most pronounced in the urban core, whereas suburban and waterfront areas more often form contiguous clusters. These patterns motivate subsequent analyses examining associations between the bird-related sentiment index and landscape and environmental variables.

**Fig 3:**
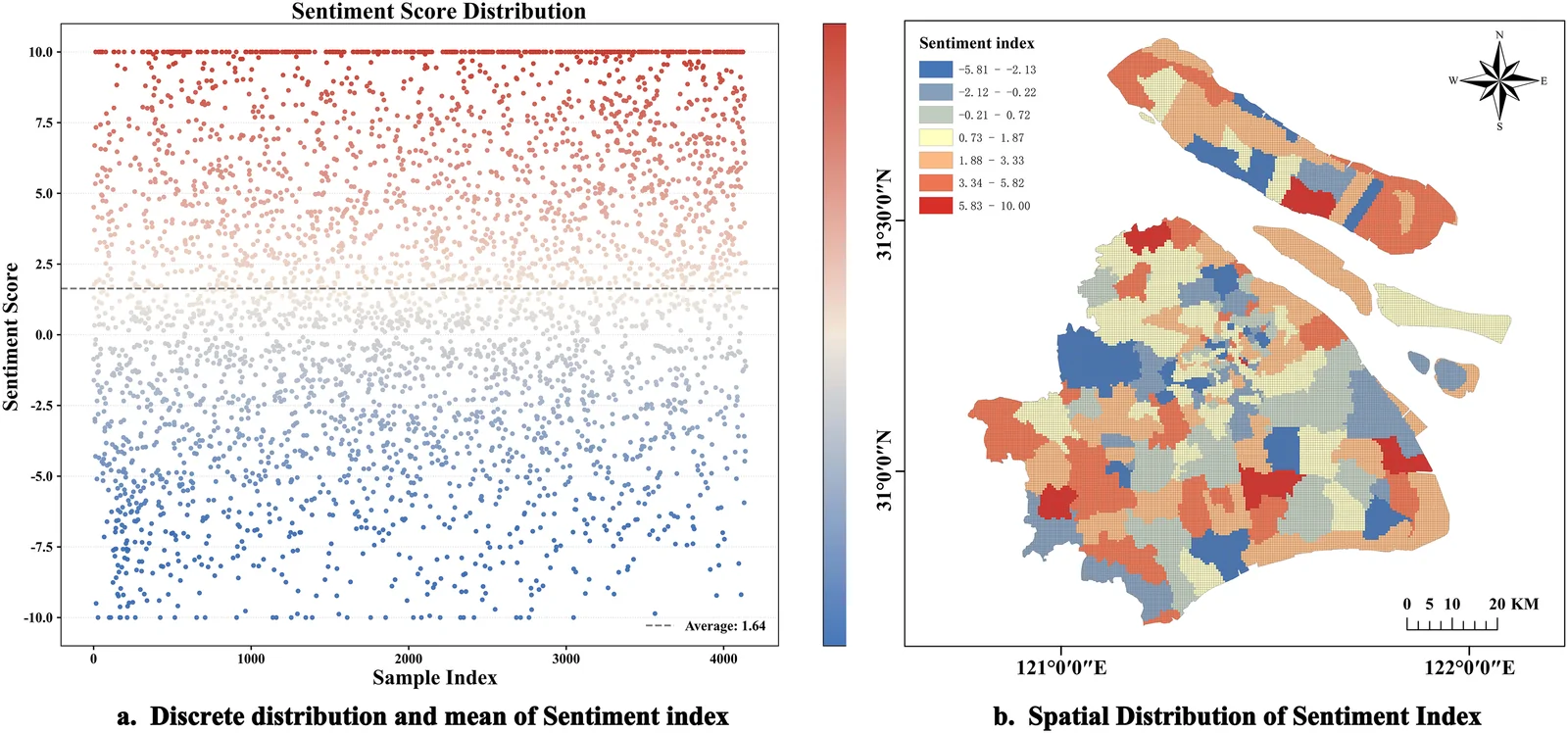
Statistical and Spatial Distribution of bird-related sentiment index. Note: The Shanghai administrative boundary in panel (b) was checked against the Shanghai administrative division standard map with approval number Hu S(2025)049 (https://shanghai.tianditu.gov.cn/map/views/standardMap.html). The sentiment distribution and thematic layers were generated by the authors using processed analytical data.

### Results of global Moran’s I, Z-scores, and p-values

Global Moran’s I tests were conducted in ArcGIS to assess the spatial autocorrelation of the bird-related sentiment index and all environmental indicators (Table 2). The results show that Moran’s I values for the bird-related sentiment index and all variables are positive, with Z-scores that are significantly positive and all passing the p < 0.001 threshold. This indicates the presence of clear positive spatial clustering across the study area rather than random spatial distributions. In terms of emotional responses, the bird-related sentiment index (I = 0.907, Z = 216.791) exhibits the most pronounced spatial structure, characterized by a high degree of clustering. Among landscape and environmental indicators, PM2.5 (0.997), AT (0.996), GSD (0.996), AP (0.992), LST (0.971), and WD (0.957) show the strongest clustering, reflecting highly stable spatial aggregation patterns. RH (0.948), PopD (0.939), and NL (0.931) also demonstrate strong spatial autocorrelation. By contrast, GSA (0.191) and Slope (0.184) have relatively low Moran’s I values, while AR (0.404) and GR (0.513) are also comparatively weaker, although still statistically significant. This suggests that the spatial clustering of these variables is more localized and dispersed in nature.

**Table 2:** Results of global Moran’s I, Z-score, and p-value.

Research Indicators	Moran’s I	Z-score	p-value
**bird-related sentiment index**	0.907	216.791	<0.001
**AT**	0.996	228.875	<0.001
**RH**	0.948	219.413	<0.001
**PM2.5**	0.997	229.715	<0.001
**NDVI**	0.876	203.202	<0.001
**LST**	0.971	231.803	<0.001
**AP**	0.992	227.961	<0.001
**AR**	0.404	96.532	<0.001
**OP**	0.569	136.055	<0.001
**GR**	0.513	122.527	<0.001
**EN**	0.604	144.410	<0.001
**WSA**	0.609	254.614	<0.001
**GSA**	0.191	48.259	<0.001
**WD**	0.957	228.541	<0.001
**GSD**	0.996	237.953	<0.001
**DEM**	0.896	198.484	<0.001
**Slope**	0.184	57.894	<0.001
**LUM**	0.541	129.196	<0.001
**PopD**	0.939	216.765	<0.001
**RND**	0.636	152.065	<0.001
**BCR**	0.597	142.682	<0.001
**NL**	0.931	222.033	<0.001

### Spatial patterns of environmental factors

Environmental variables showed clear spatial gradients across Shanghai (Fig 4; additional spatial distributions are provided in S1 Fig in S1 File). Thermal and human-activity indicators, including AT, LST, PM2.5, PopD, NL, and BCR, were generally higher in the central and intensively developed areas, whereas NDVI, GR, and OP tended to be higher in peripheral areas. Water-related indicators were concentrated mainly along riverine, waterfront, and coastal zones. These spatial patterns provide environmental context for the study area, while their associations with bird-related sentiment are examined in the subsequent analyses.

**Fig 4:**
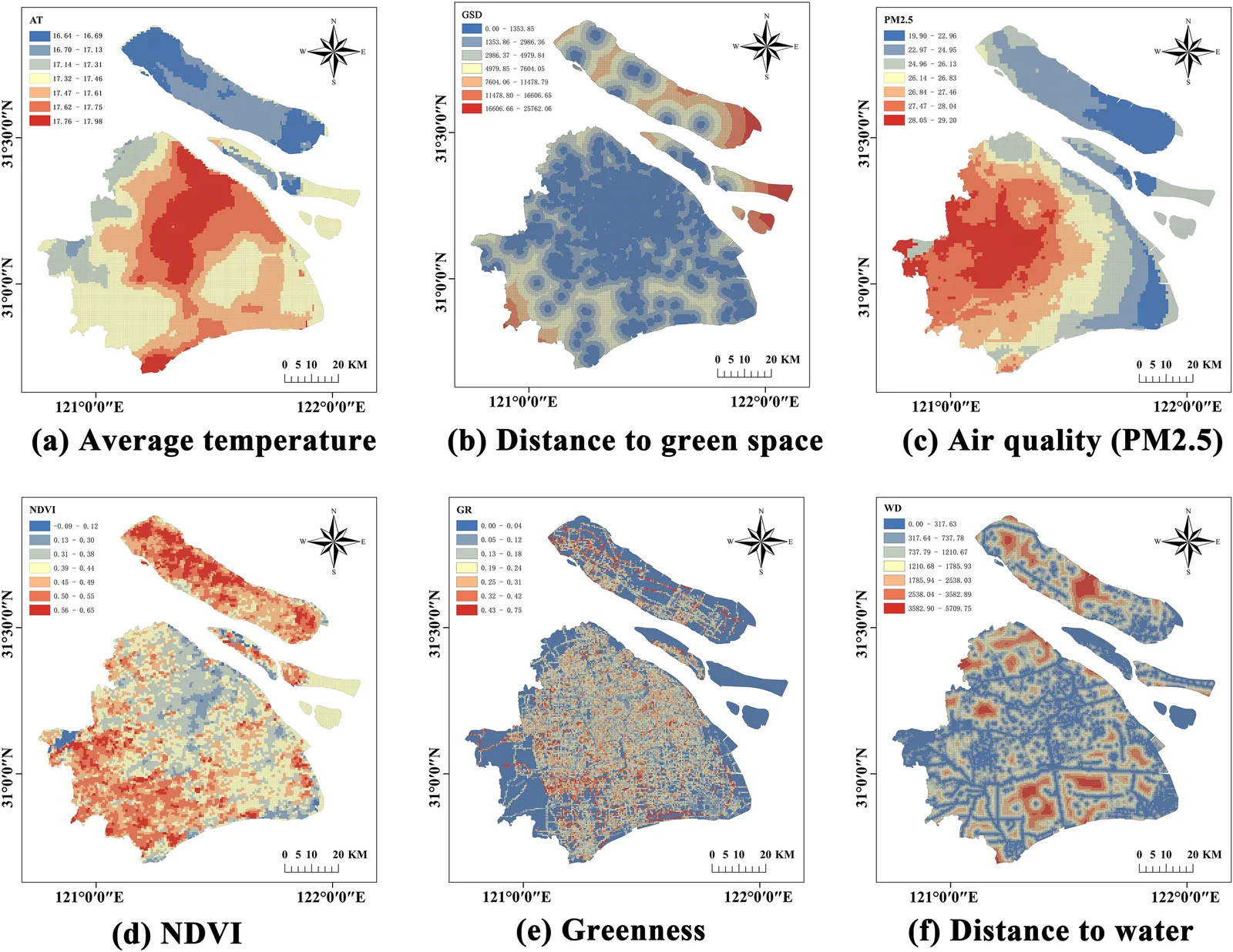
Spatial Distribution of Landscape Environmental Factors. Note: The Shanghai administrative boundary was checked against the Shanghai administrative division standard map with approval number Hu S(2025)049 (https://shanghai.tianditu.gov.cn/map/views/standardMap.html). The environmental-factor layers were generated by the authors using processed remote sensing, street-view, spatial, and statistical datasets.

### Spearman’s Rank Correlation Analysis

The Spearman rank correlation heatmap (Fig 5) shows correlation coefficients ranging from −0.83 to 0.93, indicating strong associations among landscape elements and urban activity indicators. The bird-related sentiment index exhibits generally weak monotonic correlations with most variables, but with largely consistent directions: a weak positive correlation with NDVI (ρ = 0.19); and weak negative correlations with LST (ρ= −0.31), AT (ρ= −0.27), PopD (ρ= −0.25), and NL (ρ= −0.23). PM2.5, building coverage ratio (BCR), and luminance (LUM) also show weak negative correlations. Overall, areas with better vegetation conditions are more likely to be associated with positive emotional expressions, whereas stronger human activity intensity—reflected by higher population density and nighttime light levels—is associated with slightly lower emotional scores. Water-related indicators show relatively weak associations with the bird-related sentiment index, suggesting that their relationships may depend more on specific spatial contexts. In contrast, correlations among independent variables are more pronounced. For example, NDVI–LST are moderately negatively correlated (ρ = −0.58), LST–PopD show a strong positive correlation (ρ = 0.78), and PopD–NL are also strongly correlated (ρ = 0.79). The three-dimensional perceptual indicators exhibit particularly high internal consistency (e.g., GR–EN: ρ = 0.93). Overall, the heatmap offers preliminary insight into the direction of associations among variables. It also suggests that some relationships may be nonlinear or interaction-dependent, motivating further investigation using model-based interpretability analyses.

**Fig 5:**
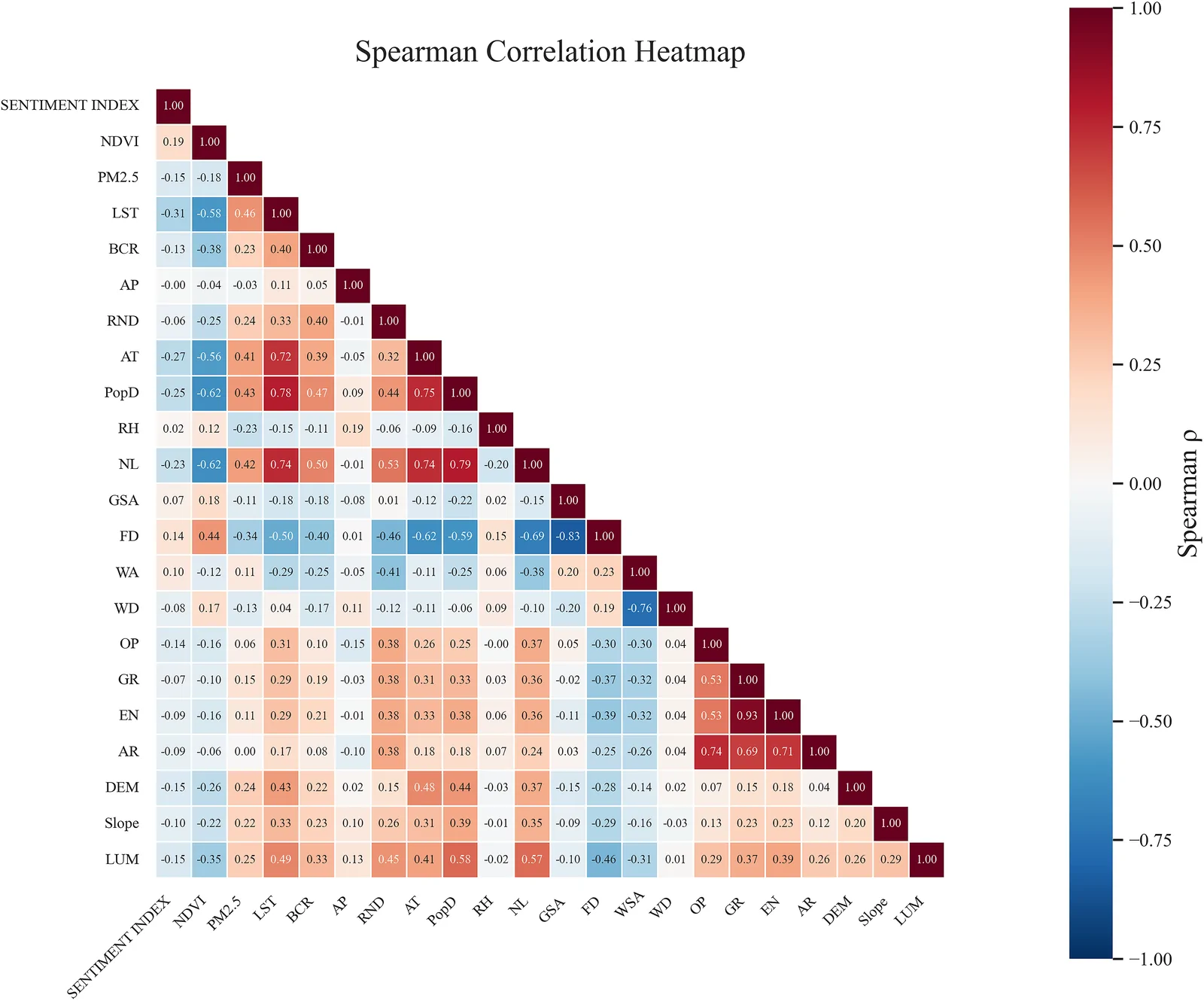
Spearman correlation heatmap.

### Nonlinear environmental associations and SHAP-based model interpretation

This section employs the SHAP method to analyze the nonlinear contribution magnitude and direction of landscape and environmental variables to model predictions of the bird-related sentiment index. The global importance results shown in the left panel of Fig 6a indicate that environmental factors dominate the model’s explanatory power. AT contributes the most (0.4738), followed by RH (0.4156), PM2.5 (0.3817), LST (0.3533), and AP (0.2548). These results suggest that model predictions of the bird-related sentiment index vary most strongly with thermal and air-quality conditions, and that these relationships involve complex nonlinear marginal contributions. GSD (0.1872), DEM (0.1689), and WD (0.1416) indicate that topographic and water-related environments also have explanatory relevance. NL (0.1081) and NDVI (0.1059) exhibit similar contributions, suggesting that human activity intensity and vegetation conditions play relatively secondary roles. In the left panel of Fig 6a, the percentages in the circular rose diagram are calculated as the proportion of each variable’s mean absolute SHAP value relative to the total mean absolute SHAP value, with sector angles allocated from 0 to 2π accordingly. The diagram confirms a high concentration of explanatory power, with the top four variables jointly accounting for 54.24% of the total contribution, indicating that a small number of key factors capture most of the model’s nonlinear variation. The beeswarm plot in the right panel of Fig 6b shows the directions of model contribution. High values of AT, PM2.5, LST, and AP predominantly exhibit negative SHAP contributions, indicating that higher temperatures, elevated pollution levels, and intense thermal environments are associated with lower predicted bird-related sentiment scores. GSD and WD also display a pattern in which high values are associated with negative contributions and low values with positive contributions. In contrast, higher NDVI values are generally associated with positive contributions, suggesting that greater vegetation cover corresponds to higher predicted bird-related sentiment scores. Most remaining variables cluster around the zero line, indicating small main effects that may act indirectly through interactions with other predictors.

**Fig 6:**
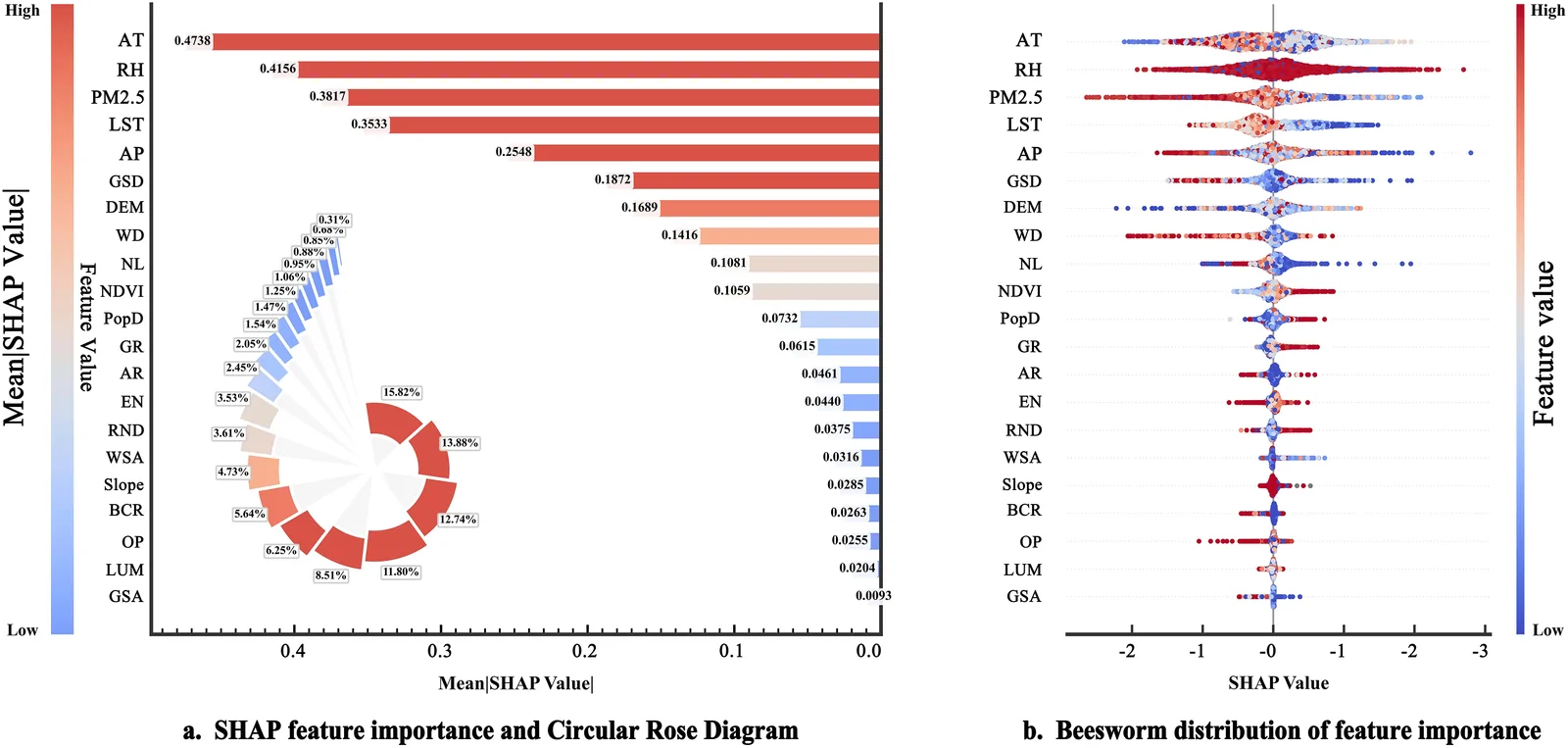
SHAP feature importance.

In the left panel of Fig 7a, the SHAP heatmap is ordered by the model output f(x), showing a gradient from negative to positive contributions. As f(x) increases, sample colors shift from blue to red, indicating that changes in predicted values coincide with coordinated changes in key environmental variables. The AT and RH bands show the strongest continuity. PM2.5 and LST also vary synchronously across multiple segments, suggesting coupled associations between thermal–humidity conditions and air quality at the sample level. AP shows stage-wise reversals in SHAP contribution, indicating a more complex and context-dependent influence. By contrast, DEM, GSD, and WD appear mainly as short, light bands, suggesting that they act primarily as local adjustments. NDVI shows an overall lighter tone, suggesting that its positive contributions are less consistent across contexts.

**Fig 7:**
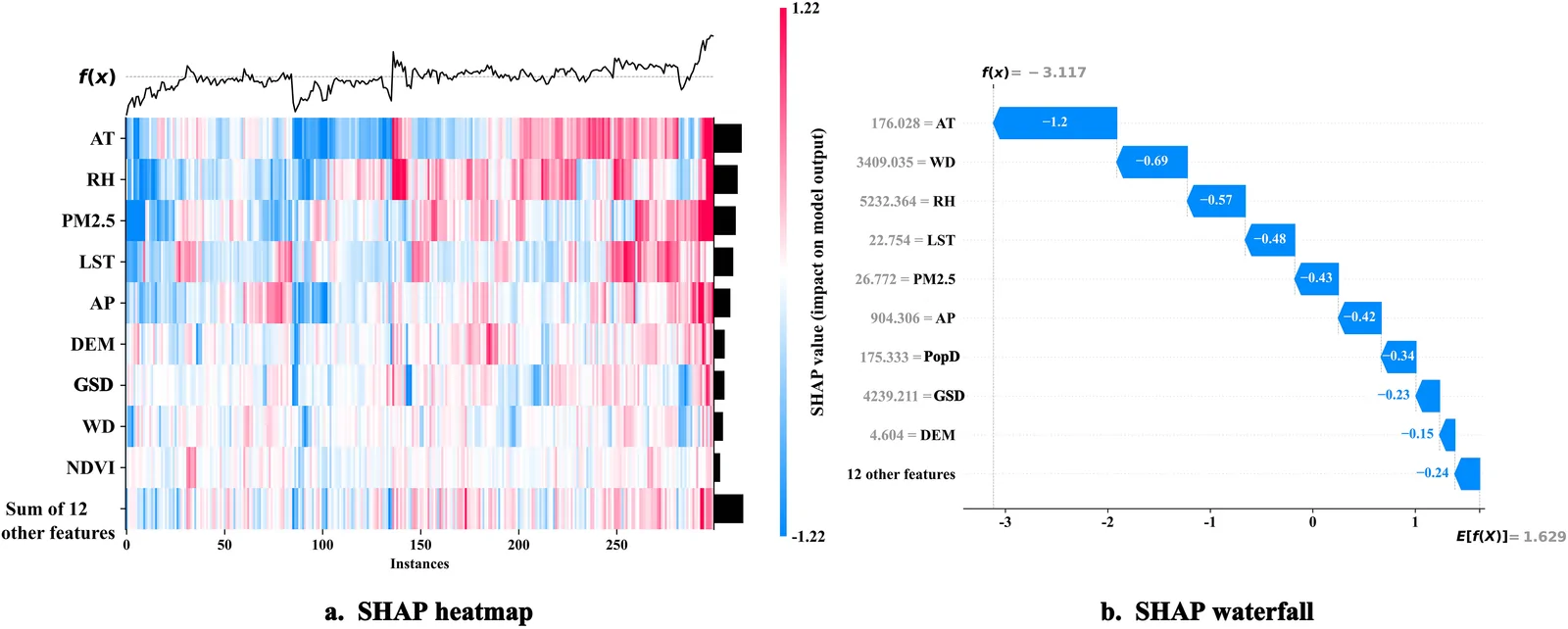
SHAP heatmap and waterfall analysis.

The waterfall plot in the right panel of Fig 7b illustrates how feature contributions accumulate nonlinearly for a representative sample. Starting from the baseline value E[f(X)]=1.629, successive negative contributions reduce the prediction to f(x)=−3.117. AT (176.028) yields the largest negative contribution (−1.20), followed by WD (−0.69), RH (−0.57), LST (−0.48), PM2.5 (−0.43), and AP (−0.42). PopD, GSD, DEM, and the remaining 12 features collectively contribute additional negative effects. This sample lacks substantial positive offsetting contributions, indicating that the combined negative contributions of thermal and air-quality variables are associated with a lower predicted bird-related sentiment score in this context.

### Spatial heterogeneity of environmental model contributions

In the GeoShapley-based spatial contribution maps, colors indicate the direction and magnitude of local SHAP contributions to the predicted bird-related sentiment index. Red represents positive contributions, blue represents negative contributions, and darker shades indicate larger contribution magnitudes. To improve readability, Fig 8 focuses on the main representative variables that are most closely related to model interpretation and planning implications. The complete spatial contribution maps and PDP results for all variables are provided in S2 Fig in S1 File.

**Fig 8:**
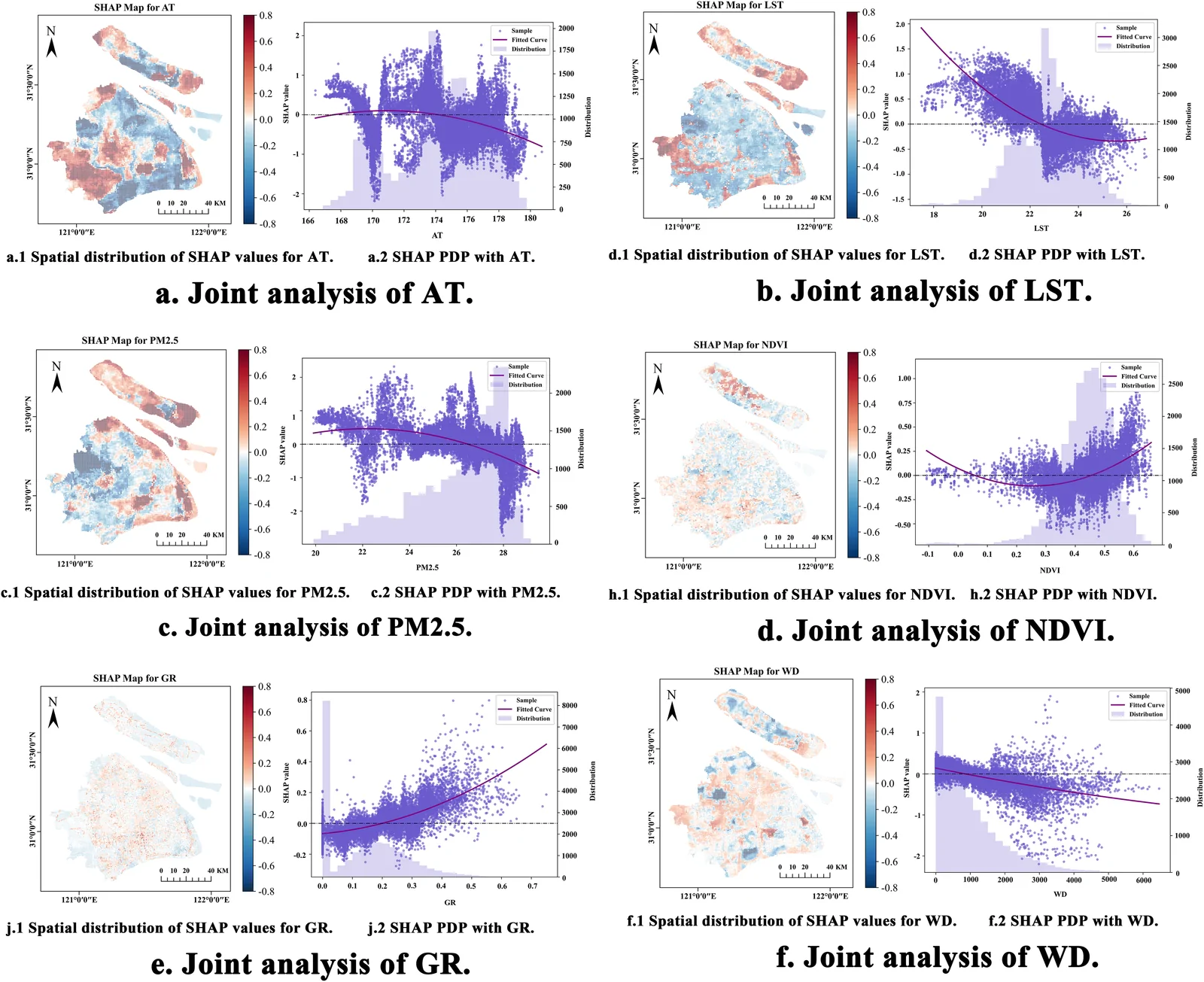
Joint analysis of SHAP partial dependence plots and GeoShapley-based spatial contribution maps. Note: The Shanghai administrative boundary in the spatial contribution maps was checked against the Shanghai administrative division standard map with approval number Hu S(2025)049 (https://shanghai.tianditu.gov.cn/map/views/standardMap.html). The SHAP partial dependence plots and GeoShapley-based spatial contribution maps were generated by the authors using processed analytical data and model outputs.

(1) Climatic and ecological factors. The partial dependence plots (PDPs) for AT and LST show overall downward trends, suggesting that higher temperatures are associated with more negative model contributions. For AT, the decline becomes evident around 17.5 and steepens after ~17.7. For LST, the steepest drop occurs around 22, and the curve begins to level off beyond ~23.5, implying a more stable negative contribution at higher temperature ranges. Spatially, negative contributions concentrate in the urban core and high-intensity built-up zones, most notably in Huangpu, Jing’an, Xuhui, Changning, Hongkou, and Yangpu. They extend toward northern Minhang, southern Baoshan, and the central–western parts of Pudong. The RH PDP is nearly flat, suggesting weak marginal contributions; its spatial contributions are correspondingly scattered. For PM2.5, SHAP values decline as concentrations increase. The decline accelerates around 28 and becomes more negative beyond ~32. Consistent with this pattern, negative contributions are more evident around the inner-ring area and core development zones of Pudong. NDVI shows a threshold-like pattern. The curve reaches a minimum around 0.28–0.30 and then rises; once NDVI exceeds ~0.45, the upward trend becomes clearer and positive contributions tend to strengthen. Accordingly, positive contributions are more common in areas with stronger ecological baselines, including Qingpu, southwestern Songjiang, Jinshan, Fengxian, and Chongming. AP shows limited overall variation, and its spatial contributions mainly appear as localized interweaving of positive and negative model contributions.
(2) Environmental landscape factors. AR shows overall positive model contributions, with positive contributions concentrated along both banks of the Huangpu River, the Suzhou Creek corridor, and coastal zones in Chongming and eastern Pudong. WSA has a relatively weak model contribution, mainly appearing as small positive contributions within waterfront areas. Both WD and GSD show negative slopes, but the declines are non-uniform. For WD, the decline is steepest between 0 and 1,000 and gradually levels off beyond ~2,000. This suggests that negative SHAP contributions associated with greater distance from water are concentrated at shorter distances. Similarly, GSD decreases rapidly between 0 and 500 and flattens beyond ~1,500, suggesting greater sensitivity to distance from green space at shorter distances. GR increases steadily with its value, with a clearer upward trend beyond ~0.30 and more stable positive contributions after ~0.40. Areas with positive GR contributions align closely with peripheral ecological zones. By contrast, GSA is generally negative or near-zero, suggesting that green space quantity alone may not correspond to higher predicted sentiment scores. Instead, spatial distribution and accessibility may be more important. EN tends to contribute negatively in inner-city areas and densely built zones of Pudong, whereas OP is more favorable in open areas such as Qingpu, Jinshan, Fengxian, and Chongming. Among topographic indicators, DEM shows overall near-zero model contributions. In contrast, slope exhibits nonlinear behavior, with more noticeable variation in areas of greater terrain re-lief, such as southwestern Songjiang.
(3) Human activity factors. LUM shows an overall negative tendency, with negative contributions concentrated in highly mixed-use, high-disturbance areas, including the inner city and core functional zones of Pudong. BCR also shows a predominantly negative pattern, with negative (blue) clusters concentrated in Huangpu, Jing’an, and Xuhui, and extending to northern Minhang, southern Baoshan, and the central–western parts of Pu-dong. NL exhibits a nonlinear relationship, with higher values tending to correspond to more negative contributions. Spatially, these patterns align with the urban core and intensively developed zones in Pudong. PopD and RND show slightly positive overall tendencies but with substantial dispersion across space. Localized positive contributions appear in accessible urban corridors, including parts of Pudong, Minhang, and Jiading, indicating pronounced spatial heterogeneity. These patterns suggest that the model contributions of PopD and RND may be context dependent and may relate to opportunities to access natural spaces and engage in bird-related activities rather than stable monotonic relationships.

## Discussion

### Spatial clustering and urban–suburban differences in bird-related emotions

Our results show that bird-related emotions are unevenly distributed across the city, forming hotspots, cold spots, and transition zones rather than a uniform pattern. Using 4,142 bird-related Weibo check-in comments (2022–2024), we constructed a bird-related sentiment index with a mean of 1.64 and a range of −10–10. Although the overall tendency is positive, the large spread indicates substantial differences in bird-related emotional experiences. Spatially, Chongming District is dominated by medium-to-high values and shows relatively continuous positive patches. In contrast, the urban core (Huangpu, Jing’an, Xuhui, Changning, Putuo, Hongkou, and Yangpu) shows rapid alternation be-tween high and low values over short distances, producing finely fragmented patches. Toward the suburban fringe, patterns become more consolidated, with relatively concentrated low-value clusters in Qingpu and Songjiang. Pudong shows a banded contrast: lower values in the central zone and more frequent medium-to-high patches along the southeastern coast and selected waterfront edges. Overall, fragmentation is strongest in the urban core, whereas suburban areas more often form contiguous patterns. Waterfront zones are also more likely to coincide with positive patches. Together, these patterns suggest that bird-related emotions reflect the combined influence of urban structure and natural resources rather than a single land-cover type. Global Moran’s I indicates strong positive spatial autocorrelation in the bird-related sentiment index (I = 0.907, Z = 216.791, p < 0.001), meaning adjacent grid cells tend to show similar emotional tendencies. This result suggests that bird-related cues, environmental comfort, and accessibility within daily activity ranges may co-vary spatially. The result is consistent with evidence from public health research suggesting that biodiversity can influence mental health through everyday perception. For example, postal-code–based studies in Canadian metropolitan areas have shown positive associations between bird and tree species diversity and better self-rated mental health, relationships that remain robust after controlling for socioeconomic factors such as income and education [8]. In Shanghai, this spatial clustering indicates relatively stable zones of higher and lower bird-related sentiment among Weibo check-in users. The fragmented pattern in the urban core is closely related to rapid street-level variations in heat, light, and crowd disturbance under high-intensity built environments, as well as to the fragmented supply of natural elements [52]. Within the same neighborhood, waterfront interfaces, pocket parks, transport corridors, and highly enclosed streets often coexist, leading to sharp short-distance fluctuations in bird cues, noise levels, and microclimates, and thus to the subdivision of emotional responses into small patches. By contrast, suburban areas or zones with stronger ecological baselines provide more stable experiential conditions, allowing emotional values to form more contiguous patterns. Correlation analyses further indicate that overall greenness alone is insufficient to explain these differences. The bird-related sentiment index shows a weak positive correlation with NDVI (ρ = 0.19), weak negative correlations with LST (ρ = −0.31), AT (ρ = −0.27), PopD (ρ = −0.25), and NL (ρ = −0.23), and even weaker associations with water-related indicators (WD: ρ = −0.08; WSA: ρ = 0.10). These results suggest that bird-related emotions are more likely shaped by a combination of accessibility, micro-environmental comfort, and perceptible visual and auditory cues. In the urban core, even where some greenery is present, bird-related sentiment may remain relatively low where access to green and blue spaces is limited and thermal stress and light pollution are higher. In contrast, where suburban ecological baselines are more continuous, bird cues are more stable, and emotional perceptions are more likely to coalesce into relatively consistent positive patches.

### Spatial heterogeneity of environmental associations

After establishing the spatial patterns, this study finds that differences in bird-related emotions are driven not merely by the amount of greenery, but by the combined effects of environmental stressors and natural accessibility. Global importance results show that explanatory power is concentrated in a small number of factors, with AT contributing the most (0.4738), followed by RH (0.4156), PM2.5 (0.3817), LST (0.3533), and AP (0.2548). The top four factors together account for 54.24% of the total contribution. These results indicate that predicted bird-related sentiment is most strongly associated with thermal conditions and air quality, consistent with previous evidence on temperature-related variation in public sentiment [53]. Natural-environment factors, meanwhile, show additional and context-dependent model contributions. This finding is consistent with evidence that the short-term mental health benefits of urban nature vary by natural type and situational context [3]. For bird-related experiences, such contextual dependence is even more pronounced because avian cues are influenced by habitat conditions, people’s willingness to linger, and whether birds can be seen and heard. Beeswarm plots and local explanations show that high values of AT, PM2.5, LST, and AP are predominantly associated with negative model contributions, indicating that higher temperatures and pollution levels are associated with lower predicted bird-related sentiment. GSD and WD display patterns in which high values tend to be negative and low values positive, suggesting that a greater distance from green spaces and water bodies is disadvantageous. In contrast, high NDVI values are more frequently associated with positive contributions. PDP and spatial contribution results further suggest that the roles of area and accessibility differ: GR improves consistently with increasing values, whereas GSA is generally negative or insignificant. This suggests that green space quantity alone does not necessarily correspond to higher predicted bird-related sentiment. Instead, accessibility and perceptible cues appear to be more important, consistent with street-level green-visibility evidence [22]. Accordingly, positive model contributions are more frequently observed where natural cues are readily accessible and perceptible, even when overall green-space coverage is not particularly high. Spatial decomposition further indicates that these relationships vary within the city, with several particularly sensitive areas. GeoShapley indicates that negative contributions from AT and LST concentrate in the urban core and high-intensity development zones, extending toward northern Minhang, southern Baoshan, and the central–western parts of Pudong. Negative PM2.5 contributions are especially pronounced around the inner ring road and in core development zones of Pudong. By contrast, positive NDVI contributions occur more often in areas with stronger ecological foundations (e.g., Qingpu, southwestern Songjiang, Jinshan, Fengxian, and Chongming). Positive AR contributions are more common along both banks of the Huangpu River, the Suzhou Creek corridor, and coastal zones in Chongming and eastern Pudong. Overall, the urban core appears more constrained by thermal stress and pollution, whereas peripheral areas generally have better natural conditions and accessibility, corresponding to more positive emotional perceptions. Waterfront areas are particularly likely to form continuous zones of positive emotional contribution. Based on these spatial patterns and prior literature, we propose two complementary explanatory pathways. The first pathway operates at the perceptual level. In grid cells with higher temperatures and pollution, emotional indices tend to show more negative contributions, and bird sounds or activity cues may be less noticeable. This interpretation is consistent with evidence that perceptions of natural soundscapes vary under different noise conditions and crowd contexts [12,14]. The second operates at the ecological level: higher thermal stress, pollution levels, and built-environment density tend to spatially coincide with negative emotional contributions, aligning with research showing that urban morphology can influence bird diversity [54].

### Bird biodiversity–friendly blue–green governance and urban regeneration strategies

Based on the identified spatial patterns and model results, blue–green governance and urban regeneration should not focus solely on increasing vegetation supply [55]. More effective entry points lie in two complementary conditions. The first is reducing the suppressive effects of thermal environments and air pollution on outdoor stay. The second is enhancing the likelihood that bird cues are perceived at places where residents are willing to linger, while minimizing disturbances, so that bird-related encounters are more likely to generate positive emotions. Central urban areas and core development zones in Pudong are more vulnerable to the adverse effects of AT, LST, and PM2.5, whereas waterfront areas often provide more stable spaces for everyday outdoor use. Accordingly, strategic emphasis should shift away from simple area expansion toward better integrating comfortable, accessible daily spaces with perceptible bird cues [56]. Specific recommendations are as follows: (1) Central urban areas and core zones of Pudong should prioritize street renewal oriented toward cooling and air purification, in order to improve walking comfort and extend dwell time. Although this study did not directly quantify urban heat-island intensity, the concentration of negative AT and LST model contributions in high-density central districts indicates clear planning relevance for urban heat-island mitigation. Cooling interventions should therefore be spatially targeted toward thermally sensitive streets and public spaces, with priority given to areas showing stronger negative thermal contributions. Priority locations include commuter arterial roads, areas around public transit stops, and pedestrian segments near schools and commercial streets. Continuous tree canopies and shading structures help reduce thermal discomfort, while permeable pavements and rain-garden facilities should be introduced along streets with high proportions of hardened surfaces to mitigate heat accumulation [56]. In poorly ventilated street segments, corner openings and façade set-backs can help create airflow corridors. Previous studies show that street tree canopy coverage is associated with improved pedestrian thermal comfort and increased willingness to walk, providing an evidence base for prioritizing such interventions [57]. It should be noted that tree-canopy coverage was not directly quantified as an independent predictor in this study. However, the contrasting patterns observed for NDVI, street-level Greenness (GR), and Green Space Area (GSA) suggest that vegetation configuration and street-level perceptibility may warrant greater planning attention than simply increasing total green-space area. Once thermal stress and pollution burdens are reduced, residents are more likely to linger and observe their surroundings; bird songs and avian activities thus become easier to notice and more likely to elicit positive emotions. (2) Waterfront spaces along the Huangpu River and Suzhou Creek should be enhanced in terms of continuity and accessibility, allowing blue–green corridors to function as everyday settings for residents’ encounters with bird cues. Fragmented shorelines and gaps in green belts can be incrementally repaired by completing pedestrian and cycling paths, and by increasing the density of safe crossings and access points near bridgeheads, tributary confluences, and major intersections, thereby forming a more continuous slow-mobility network. At river-network nodes, small-scale stopping points with clear wayfinding can be introduced to encourage residents to pause, observe, and listen without entering ecologically sensitive areas. Research demonstrating the role of waterfront green spaces in supporting urban bird communities can serve as an ecological justification [58]. On this basis, continuous and accessible waterfront slow-mobility systems are more likely to increase repeated everyday exposure to bird cues, leading to cumulative positive emotional experiences. (3) Community green spaces and street-corner greens should prioritize enhancing the perceptibility of bird cues rather than simply expanding the area. Pocket parks can adopt layered vegetation structures (trees, shrubs, and ground cover), incorporate ecotones, increase flowering or fruit-bearing shrubs, retain limited areas of low-frequency–mowed grass, and install shallow drinking water points or small wetland flowerbeds to increase the likelihood of bird presence and activity. In highly enclosed street segments, reducing continuous hard edges and introducing green openings and small resting spaces may enhance visual and auditory perception while reducing disturbance to birds [11]. (4) Zoned urban–suburban governance, combined with participatory programs, can further integrate bird encounters into residents’ daily lives. In areas with stronger ecological foundations (e.g., Qingpu, Songjiang, Fengxian, Jinshan, Chongming, and coastal zones), management should prioritize maintaining ecological networks and adopting low-disturbance strategies. Guided routes and visitor-flow redistribution can reduce pressure on sensitive sites and support stable bird populations. In central urban areas, efforts should focus on increasing contact frequency by using community green corridors, pocket parks, and waterfront slow-mobility networks as everyday settings for bird encounters. Low-intensity bird-watching and educational programs can be held at sites with adequate carrying capacity, accompanied by clear behavioral guidelines to minimize disturbance. In addition, social media can serve not only as a source of data on public emotions and nature experiences, but also as a complementary channel for urban bird-related environmental education and public engagement. Accessible online content on locally common bird species, their basic ecological roles, and responsible birdwatching practices could complement in-person activities and further support participatory bird monitoring and citizen-science initiatives. Embedding contact opportunities in daily spaces may also enhance public health benefits. Experimental evidence suggests that birdwatching is associated with higher subjective well-being and lower psychological distress [33].

### Research limitations and future directions

Using Shanghai as a case study, we characterize within-city spatial variation in public emotions expressed in bird-related discussions and examine associations with blue–green spaces and built-environment characteristics. Because cities differ in development stage, climatic context, and social media usage patterns, these findings may not be directly generalizable to other cities. This caution aligns with the careful framing adopted in case-based studies using short-video platforms [59]. Future research could therefore conduct parallel or comparative studies across cities in different climate zones, of varying sizes, and at different development levels, to test the robustness of these findings and to identify the contextual conditions under which divergences emerge. From a data perspective, Weibo users are subject to selection biases in age, education, and occupation. Because Weibo check-in comments are posted by active platform users, the results should be interpreted as the bird-related sentiment patterns of active Weibo check-in users in Shanghai rather than as a complete representation of all residents’ perceptions. Such data may overrepresent younger users, recreational visitors, and people visiting popular parks, waterfronts, wetlands, or other high-visibility locations, while underrepresenting residents who do not use Weibo or rarely post check-in comments. In addition, some areas generate few posts, creating information gaps. These gaps are more likely to reflect variation in platform activity than a true absence of bird-related encounters. Moreover, text-based data lack systematic socioeconomic and health information, making it difficult to assess how different social groups respond to bird cues. This limitation echoes broader discussions on unequal access to human–nature interactions [60]. Future studies could integrate surveys, demographic data, and participatory birdwatching records. Incorporating gender, age, income, and health status would enable analyses of group differences from a social stratification perspective. Methodologically, the indicators used in this study have not yet been cross-validated against measured bird diversity or acoustic monitoring data. Previous research suggests that perceived biodiversity is correlated with, but not equivalent to, measured biodiversity [9,61]. Future work could therefore combine emotional spatial patterns with citizen science records, automated acoustic data, and habitat indicators, and compare their respective influences at a unified spatial scale. In addition, the bird-related sentiment index and several environmental predictors showed significant spatial autocorrelation, which may influence model validation based on random training–testing splits. In spatially structured data, neighboring samples often share similar environmental conditions and response patterns; therefore, random splitting may place spatially adjacent grids in both the training and testing sets, reducing the spatial independence of testing samples and leading to optimistic estimates of model performance [62]. In this study, the modeling results are mainly used to examine associations between environmental variables and the bird-related sentiment index, rather than to claim spatially independent predictive accuracy. Future research could further apply spatial block validation to test model transferability when training and testing samples are spatially separated [63]. Temporal extensions could further distinguish migratory, breeding, and wintering seasons, as well as weekday versus weekend and daytime versus nighttime patterns. Finally, future research could incorporate species-level identification to compare public emotional responses to different bird species. It would also be valuable to examine whether familiarity with local bird species influences appreciation and emotional responses, and whether the conservation status of locally distinctive, endemic, threatened, or endangered species is associated with stronger positive sentiment, place attachment, or local pride. Comparing public perceptions of native and non-native species could further support more targeted environmental education, species conservation, and spatial design interventions.

## Conclusion

Using Shanghai as a case study, this research integrates Weibo data with spatial information and street-level environmental features to analyze the bird-related sentiment index and its relationship with the urban environment at a 500 m × 500 m grid scale. The results show that the mean bird-related sentiment index is 1.64, indicating an overall positive sentiment tendency among Weibo check-in users in Shanghai, but with pronounced spatial heterogeneity. Chongming District shows relatively high sentiment scores (1.88–10.00). By contrast, the urban core exhibits interwoven high and low values, with greater variability and a score range of −2.12 to 1.87. Qingpu and Songjiang emerge as inland low-sentiment zones (−5.81 to −2.13), whereas waterfront and coastal areas form distinct belts of medium-to-high values (1.88–10.00). Among the predictors, AT, LST, and PM2.5 show the strongest negative associations with the bird-related sentiment index, with mean absolute SHAP values of 0.4738, 0.3533, and 0.3817, respectively. Natural-environment indicators, particularly NDVI and GR, are generally associated with higher sentiment scores. NDVI shows clearer positive contributions when values exceed ~0.45, whereas GSA shows weak overall contributions and is near-zero or negative in most ranges. Spatially, the urban core is more often characterized by negative contributions from AT and LST, while peripheral and waterfront areas more frequently show positive contributions from NDVI, water-related indicators, and accessibility-related factors. These findings are based on bird-related Weibo check-in comments in Shanghai and should be interpreted as observational associations rather than causal evidence. They are not necessarily generalizable to all residents or other cities. Within this scope, three planning priorities emerge from the results. First, central and intensively developed areas should prioritize the mitigation of thermal stress and air pollution, with cooling and urban heat-island mitigation measures targeted toward streets and public spaces showing stronger negative thermal contributions. Second, the continuity and accessibility of waterfront areas and green corridors should be improved to increase everyday opportunities for contact with blue–green spaces. Third, community green spaces, street corners, and pedestrian environments should enhance the perceptibility of vegetation and provide small-scale resting spaces that support everyday encounters with birds. Overall, these findings suggest that biodiversity conservation and urban well-being can be better aligned when ecological resources, environmental comfort, and daily accessibility are considered together.

## Supporting information

S1 File Supporting figures and tables(continued). This file includes supplementary figures and tables supporting the data processing, spatial analysis, model validation, and machine-learning interpretation procedures used in this study.
(DOCX)
