# Supplementary material for: Spatial patterns of the bird-related sentiment index and its nonlinear environmental associations: Evidence from social media and explainable machine learning
Source: PLoS One. 2026 Nov 10;21(11):e0360060. doi: 10.1371/journal.pone.0360060 (PMC13750583; doi:10.1371/journal.pone.0360060)
Supplement: Supplementary file 1 [file pone.0360060.s001.docx]

**S1 File. Supporting figures and tables.**

**S1 Fig. Spatial Distribution of Landscape Environmental Factors.** Note: The Shanghai administrative boundary was checked against the Shanghai administrative division standard map with approval number Hu S(2025)049 (https://shanghai.tianditu.gov.cn/map/views/standardMap.html). The environmental-factor layers were generated by the authors using processed remote sensing, street-view, spatial, and statistical datasets.

**S2 Fig. Joint analysis of SHAP partial dependence plots and GeoShapley-based spatial contribution maps.** Note: The Shanghai administrative boundary in the spatial contribution maps was checked against the Shanghai administrative division standard map with approval number Hu S(2025)049 (https://shanghai.tianditu.gov.cn/map/views/standardMap.html). The SHAP partial dependence plots and GeoShapley-based spatial contribution maps were generated by the authors using processed analytical data and model outputs.

**S1 Table. Spatial matching and grid-level distribution of valid Weibo comments at the 500 m × 500 m scale.**

| **Category** | **Item** | **Value** |
| --- | --- | --- |
| Spatial matching | Total valid bird-related Weibo comments | 4,142 |
| Spatial matching | Comments successfully assigned to the 500 m × 500 m grid system | 4,011 |
| Spatial matching | Spatial matching rate | 96.84% |
| Grid coverage | Total 500 m × 500 m grid cells in the Shanghai study area | 29,273 |
| Grid coverage | Grid cells with at least one valid Weibo comment | 1,280 |
| Grid-level sample size | Average number of comments per occupied grid | 3.13 |
| Grid-level sample size | Grid cells with no fewer than 3 comments | 310 |
| Grid-level sample size | Grid cells with no fewer than 5 comments | 157 |
| Distribution across occupied grids | 1–2 comments per grid | 970 |
| Distribution across occupied grids | 3–4 comments per grid | 153 |
| Distribution across occupied grids | ≥5 comments per grid | 157 |
| Distribution across occupied grids | Total occupied grids | 1,280 |

**Note:** Occupied grids refer to 500 m × 500 m grid cells containing at least one valid bird-related Weibo comment. Grids without valid comments were not assigned artificial zero or neutral sentiment values.

**S2 Table. Detailed data-flow and screening rules for Weibo bird-related check-in comments from 2022 to 2024.**

| **Step** | **Procedure** | **Detailed screening rule or description** | **2022** | **2023** | **2024** | **Total** |
| --- | --- | --- | --- | --- | --- | --- |
| 1 | Initial keyword retrieval | Weibo check-in comments were retrieved within Shanghai from January 1, 2022 to December 31, 2024 using a broad set of bird-related Chinese-language search terms, which were translated into English for reporting. The keyword set covered: bird, flying bird, little bird, migratory bird, fledgling, juvenile bird, birdwatching, bird photography, bird protection, bird-loving, release of captive animals, pigeon, sparrow, magpie, turtledove, swallow, crow, myna, hwamei, light-vented bulbul, hoopoe, swan, white swan, black swan, wild duck, mallard, ruddy shelduck, mandarin duck, goose, wild goose, great egret, night heron, Chinese pond heron, grey heron, heron, seagull, gull, cormorant, eagle, falcon, hawk, raptor, owl, scops owl, woodpecker, kingfisher, thrush, swift, bulbul, chicken, duck, domestic goose, and quail. Broad terms such as chicken, duck, domestic goose, and quail were retained at the initial retrieval stage to avoid missing potentially relevant bird-related posts, but they were manually screened later because they could also refer to food or other non-research contexts. | 10,044 retained | 13,220 retained | 25,331 retained | 48,595 retained |
| 2 | Core field check | Core analytical fields were checked, including Weibo text, posting time, Weibo post ID, longitude, and latitude. Records with missing text, missing posting time, missing post ID, or missing coordinates would have been removed. | 1 removed; 10,043 retained | 3 removed; 13,217 retained | 2 removed; 25,329 retained | 6 removed; 48,589 retained |
| 3 | Exact duplicate check | Exact duplicates were examined using Weibo post IDs. Records with duplicated post IDs would have been removed to prevent repeated posts from entering the analysis. | 0 removed; 10,043 retained | 1 removed; 13,216 retained | 0 removed; 25,329 retained | 1 removed; 48,588 retained |
| 4 | Coordinate validity check | Coordinates were checked against the Shanghai study area. Records with missing, abnormal, or clearly out-of-study-area coordinates would have been excluded. Although some records lacked place names or detailed addresses, their longitude and latitude were valid and therefore could still be assigned to 500 m × 500 m grids. | 0 removed; 10,043 retained | 0 removed; 13,216 retained | 0 removed; 25,329 retained | 0 removed; 48,588 retained |
| 5 | Manual thematic relevance screening | Records were manually screened according to predefined inclusion and exclusion criteria. Posts were retained when they described direct or place-based outdoor urban experiences related to birds, bird sounds, bird flocks, bird activity traces, birdwatching, bird photography, or bird-related disturbance. Posts were removed when bird-related words appeared only in food or dish names, brand names, shop names, place names, internet homophones, metaphorical expressions, pet-bird or indoor cage-bird discussions, advertisements, reposts without original experiential content, purely informational notices, or texts without clear outdoor bird-related experience. | 9,333 removed; 711 retained | 12,024 removed; 1,196 retained | 23,096 removed; 2,235 retained | 44,453 removed; 4,142 retained |
| 6 | Final analytical sample | The final sample included only Weibo check-in comments with valid Shanghai coordinates and clear outdoor bird-related experiential content. These records were used for sentiment analysis, grid-level aggregation, spatial analysis, and machine-learning modeling. | 711 retained | 1,196 retained | 2,235 retained | 4,142 retained |

**S3 Table. Validation performance of the ERNIE + LSTM–CNN sentiment classification model based on manually annotated bird-related Weibo comments.**

| **Sentiment class** | **Precision** | **Recall** | **F1-score** | **Support** |
| --- | --- | --- | --- | --- |
| **Negative** | 0.838 | 0.944 | 0.887 | 142 |
| **Neutral** | 0.867 | 0.833 | 0.850 | 78 |
| **Positive** | 0.943 | 0.893 | 0.917 | 280 |
| **Macro average** | 0.883 | 0.890 | 0.885 | 500 |
| **Weighted average** | 0.901 | 0.898 | 0.898 | 500 |
| **Overall accuracy** | — | — | 0.898 | 500 |

**S4 Table. Confusion matrix for the manually annotated bird-related Weibo validation subset.**

| **Manual annotation / Model prediction** | **Negative** | **Neutral** | **Positive** | **Total** |
| --- | --- | --- | --- | --- |
| Negative | 134 | 2 | 6 | 142 |
| Neutral | 4 | 65 | 9 | 78 |
| Positive | 22 | 8 | 250 | 280 |
| Total | 160 | 75 | 265 | 500 |

**S5 Table. Data sources, calculation formulas, and variable abbreviations used in this study.**

| **Type** | **Dimension** | **Research indicators** | **Definitions and formulas** | **Quantitative methods** |
| --- | --- | --- | --- | --- |
| **Climate & ecological factors** | 2D | Average temperature（AT） | The raster dataset of monthly mean temperature was obtained from <https://www.geodata.cn>. | ArcMap |
|  |  | Relative humidity (RH) | The monthly average relative humidity raster dataset was obtained from <https://www.geodata.cn>. | ArcMap |
|  |  | Air quality (PM2.5) | The global annual PM2.5 dataset with a 1 km spatial resolution was obtained from <https://www.geodata.cn>. | ArcMap |
|  |  | Normalized Difference Vegetation Index（NDVI） | Sentinel-2 L2A imagery was obtained via Google Earth Engine. | ArcMap |
|  |  | Land surface temperature (LST) | The Landsat 8 OLI_TIRS satellite data were obtained from <https://www.gscloud.cn>. | ArcMap |
|  |  | Average precipitation（AP） | The monthly average precipitation raster dataset was obtained from <https://www.geodata.cn>. | ArcMap |
| **Environmental factors** | 3D | Aquatic rate (AR) | The proportion of water bodies in the image  $A=\frac{P_{aquatic}}{P_{total}}\times100\%$  Where $P_{aquatic}$is the total number of water-related pixels identified in the image, and$P_{total}$is the total number of recognized pixels in the image. | Mask2Former |
|  |  | Openness (OP) | The proportion of sky in the image  $O=\frac{P_{sky}}{P_{total}}\times100\%$  Where $P_{sky}$ is the total number of sky-related pixels (e.g., blue or white sky) identified through semantic segmentation in the image, and $P_{total}$ is the total number of recognized pixels in the image. | Mask2Former |
|  |  | Greenness (GR) | The proportion of green plants in the image  $G=\frac{P_{green}}{P_{total}}\times100\%$  Where $P_{green}$ refers to the total number of pixels classified as vegetation, including grass, shrubs, and trees, based on semantic segmentation; $P_{total}$ represents the total pixel count of the recognized image area. | Mask2Former |
|  |  | Enclosure (EN) | The proportion of architectural enclosure in the image  $E=\frac{\sum_{i=1}^{2} (B_{i}+T_{i}+W_{i}+F_{i})}{\sum_{i=1}^{2} (1-SP_{i})}$  In the formula, $B_{i}$ , $T_{i}$, $W_{i}$, and $F_{i}$ respectively indicate the proportions of pixels belonging to buildings, trees, walls, and fences. | Mask2Former |
|  | 2D | Water space area (WSA) | The global Water space area was obtained from <https://www.globallandcover.com>. | ArcMap |
|  |  | Green space area (GSA) | The global land cover dataset was obtained from <https://www.globallandcover.com>. | ArcMap |
|  |  | Distance to water (WD) | The global distance to water was obtained from <https://www.globallandcover.com>. | ArcMap |
|  |  | Distance to green space (GSD) | The global distance to green space was obtained from <https://www.globallandcover.com>. | ArcMap |
|  |  | DEM | The global DEM was obtained from  [SRTM Data – CGIAR-CSI SRTM](https://srtm.csi.cgiar.org/srtmdata/) | ArcMap |
|  |  | Slope | The global Slope was obtained from  <https://www.globallandcover.com>. | ArcMap |
| **Human factors** | 2D | Land use mix (LUM) | An indicator assessing the spatial and functional integration of different land use types.  $LUM=-\sum(P_{i}\cdot logP_{i})$  where$P_{i}$represents the proportion of the *i*-th land use type within the total area of the spatial unit. | ArcMap |
|  |  | Population density  (PopD) | Population in each research unit. | ArcMap |
|  |  | Road network density  (RND) | Total length of roads within the research unit.  Road network data sourced from  <https://www.openstreetmap.org/>. | ArcMap |
|  |  | Building coverage ratio (BCR) | The proportion of the total building footprint area to the overall grid area.  $BCR=\frac{B}{A}$  where$B$represents the total footprint area of all buildings within the unit (km²), and$A$refers to the total land area of the corresponding spatial unit (km²).  Building density data sourced from  <https://www.openstreetmap.org/>. | ArcMap |
|  |  | Nighttime light pollution（NL） | The global “NPP-VIIRS-like” nighttime light dataset with a 500 m spatial resolution was obtained from <https://www.geodata.cn>. | ArcMap |

**S6 Table. Summary of street-view image collection and semantic segmentation processing.**

| **Item** | **Description** |
| --- | --- |
| Road data source | OpenStreetMap (OSM) road network |
| Street-view image source | Baidu Street View API |
| Street-view data collection and processing time | March 2025 |
| Main acquisition years of Baidu Street View imagery | Predominantly 2022–2023 |
| Sampling software | ArcMap |
| Sampling interval | 50 m along the OSM road network |
| Number of street-view sampling points | 471,839 |
| Viewing directions per sampling point | Four fixed headings: 0°, 90°, 180°, and 270° |
| Number of street-view images | 1,887,356 |
| Image stitching | Multi-directional images were stitched into panoramic views using OpenCV. OpenCV version 4.10.0 was used for image stitching. |
| Image quality-control screening | Images with inaccessible links, duplicated views, severe blur, heavy occlusion, abnormal viewing angles, or clearly abnormal segmentation outputs were excluded from indicator calculation |
| Semantic segmentation model | Mask2Former |
| Model source / training dataset | Pretrained semantic segmentation model trained on the Mapillary Vistas dataset |
| Main recognized street-scene categories | Vegetation, sky, buildings, road surfaces, water-related elements, and other street elements |
| Derived streetscape indicators | Greenness, Openness, Enclosure, and Aquatic Rate |
| Indicator calculation | Pixel proportions of recognized categories were calculated from segmentation outputs |
| Segmentation reliability | Supported by the reported benchmark performance of Mask2Former and image-level quality-control screening |
| Spatial aggregation scale | 500 m × 500 m grid cells |
| Final use in this study | Grid-level streetscape indicators were linked with the bird-related sentiment index for spatial analysis and machine-learning modeling |

**S7 Table. Final hyperparameters of the XGBoost model.**

| **Hyperparameter** | **Value** |
| --- | --- |
| n_estimators | 900 |
| max_depth | 6 |
| learning_rate | 0.035 |
| min_child_weight | 2 |
| subsample | 0.82 |
| colsample_bytree | 0.88 |
| reg_alpha | 0.05 |
| reg_lambda | 1.0 |
| random_state | 123 |
